# Inhibition of TGF-β signaling supports high proliferative potential of diverse p63^+^ mouse epithelial progenitor cells *in vitro*

**DOI:** 10.1038/s41598-017-06470-y

**Published:** 2017-07-20

**Authors:** Daisuke Suzuki, Filipa Pinto, Makoto Senoo

**Affiliations:** Department of Molecular and Cell Biology, Boston University Henry M. Goldman School of Dental Medicine, Boston, MA 02118 USA

## Abstract

Mouse models have been used to provide primary cells to study physiology and pathogenesis of epithelia. However, highly efficient simple approaches to propagate mouse primary epithelial cells remain challenging. Here, we show that pharmacological inhibition of TGF-β signaling enables long-term expansion of p63^+^ epithelial progenitor cells in low Ca^2+^ media without the need of progenitor cell-purification steps or support by a feeder cell layer. We find that TGF-β signaling is operative in mouse primary keratinocytes in conventional cultures as determined by the nuclear Smad2/3 localization. Accordingly, TGF-β signaling inhibition in crude preparations of mouse epidermis robustly increases proliferative capacity of p63^+^ epidermal progenitor cells, while preserving their ability of differentiation in response to Ca^2+^ stimulation. Notably, inhibition of TGF-β signaling also enriches and expands other p63^+^ epithelial progenitor cells in primary crude cultures of multiple epithelia, including the cornea, oral and lingual epithelia, salivary gland, esophagus, thymus, and bladder. We anticipate that this simple and efficient approach will facilitate the use of mouse models for studying a wide range of epithelia by providing highly enriched populations of diverse p63^+^ epithelial progenitor cells in quantity.

## Introduction

Homeostasis and regeneration of epithelia are maintained by self-renewal, proliferation, and differentiation of tissue-specific stem cells^1–3^. The development of 3T3-J2 feeder cell co-culture by Rheinwald and Green^4^ has allowed expansion of human primary keratinocytes, and later corneal epithelia, and contributed to not only our understanding of epithelial cell biology but also therapeutic strategies in regenerative medicine^5–7^. 3T3-J2 cells support the proliferative potential of epithelial progenitor cells in a paracrine manner^8, 9^ and have been used to assess self-renewal capacity of p63^+^ epithelial stem cells^10–12^. The transcription factor p63 plays an essential intrinsic role in regulating stem cell self-renewal^12, 13^. Indeed, mice lacking p63 show loss or severe hypoplasia in all epithelia that normally express p63 in their stem cell compartments, such as the skin, cornea, thymus, salivary gland, esophagus, mammary gland, prostate, and bladder^14, 15^. Thus, p63 plays a key role in maintaining the proliferative capacity of epithelial progenitor cells of diverse epithelia.

Mouse models have been used in studies of normal and disease conditions of epithelia. However, the growth of p63^+^ mouse primary epithelial cells (e.g. primary keratinocytes) in culture rapidly declines and the cells become terminally differentiated^16^, a limit that restricts the use of primary cells for functional analyses of epithelia. As proliferation and differentiation of epithelial cells are tightly coupled through the induction of cyclin-dependent kinase (CDK) inhibitor genes^17–19^, suppression of growth arrest and of premature differentiation are both potential approaches to improve the lifespan of p63^+^ mouse primary epithelial progenitor cells^17, 20–23^. Although the use of low calcium (Ca^2+^) media has extended proliferation of p63^+^ mouse epithelial progenitor cells short term^16, 24^, the most effective protocols to propagate these primary cells rely on modified 3T3-J2 feeder co-culture^20, 22^ or the use of fluorescence activated cell sorting (FACS)-purified progenitor populations with triple drug inhibitors^23^. Highly efficient protocols that eliminate the use of undefined factors (e.g. feeder cells), labor-intensive purification steps, and the potentially complex effects of multiple drugs will facilitate the use of primary epithelial cells of mice for studying the physiology and pathogenesis of p63-dependent epithelia.

Transforming growth factor-β (TGF-β) signaling regulates proliferation and differentiation of many different progenitor cells, including those that are controlled by p63^25^. TGF-β signaling is mediated through the receptor complex consisting of the Type I TGF-β receptor (TGFβR1/ALK5) and the Type II TGF-β receptor (TGFβR2)^26^. Upon binding of the TGF-β ligands, TGFβR2 phosphorylates and activates TGFβR1/ALK5, resulting in the phosphorylation and nuclear translocation of Smad2/3, downstream effectors of TGF-β signaling^27^. Consistent with its growth suppressive function, phosphorylated Smad2/3 are barely detectable in basal cells in many different epithelia in mouse, including the skin^23^. Notably, however, we find that a large fraction of Smad2/3 is localized in the nuclei of mouse primary epidermal keratinocytes in a growth-permissive low Ca^2+^ condition that would ordinarily support keratinocyte proliferation. We hypothesize that inhibition of TGF-β signaling would suppress Smad2/3 nuclear localization and consequently increase the proliferative capacity of p63^+^ mouse primary epithelial cells *in vitro*.

In this report, we show that the use of a single small molecule inhibitor of TGF-β signaling in crude mouse tissue preparations enriches p63^+^ epithelial progenitor cells and supports their long-term proliferative capacity in a low Ca^2+^ condition. This methodology can produce a large quantity of diverse p63^+^ mouse epithelial progenitor cells without the need of a feeder layer or progenitor cell-purification steps. We anticipate that this simple and efficient approach will facilitate the use of mouse models for studying a wide range of p63-dependent epithelia.

## Result

### Inhibition of TGF-β signaling stimulates the proliferation of mouse primary epidermal cells *in vitro*

**Figure 1:**
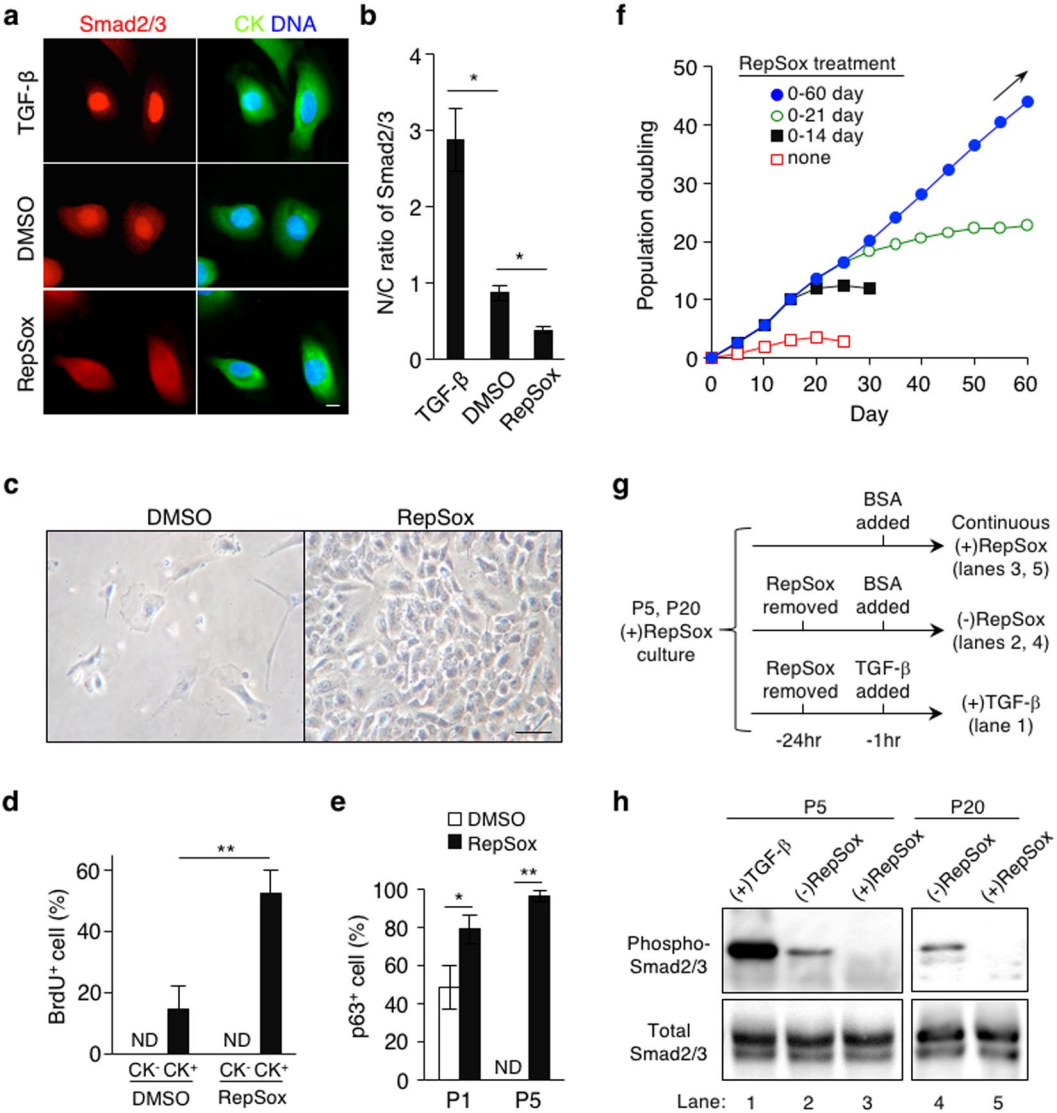
Inhibition of TGF-β signaling enables long-term expansion of p63^+^ mouse primary epidermal progenitor cells *in vitro*. (**a**) Subcellular localization of Smad2/3 in newborn mouse primary epidermal keratinocytes grown in CnT-PR media. The cells were stimulated with 1 ng/ml TGF-β2, 0.1% DMSO (as a control) or 1 μM RepSox for 1 hr. *CK*, pan-cytokeratin; *DNA*, nuclear counterstaining with Hoechst 33342; Bar = 10 μm. (**b**) Nuclear-to-cytoplasmic (N/C) ratios of Smad2/3 as determined by fluorescence intensity. Data shown are mean ± s.e.m. (n = 6). *P < 0.05. (**c**) Representative images of primary cells of newborn mouse epidermis at day 14 of culture in the presence or absence of 1 μM RepSox. Bar = 25 μm. (**d**) A short-pulse labeling with BrdU in primary culture of newborn mouse epidermal cells in the presence or absence of 1 μM RepSox. Data shown are % per field, expressed as mean ± s.e.m. (n = 4). *ND*, not detected; **P < 0.01. (**e**) Percentages of p63^+^ cells at passage 1 and 5 (P1 and P5, respectively) grown in the presence (solid) or absence (open) of 1 μM RepSox. Data shown are % per field, expressed as mean ± s.e.m. (n = 3 and 4 for P1 and P5, respectively). *ND*, not detected; *P < 0.05; **P < 0.01. (**f**) Population doubling of newborn mouse-derived, RepSox-expanded P2 epidermal cells grown in CnT-PR media in the presence of 1 μM RepSox for 0, 14, 21, and 60 days. Data shown are representative of three independent experiments with similar results. Arrow indicates continuous cell growth. (**g**,**h**) Removal of RepSox associates with increased Smad2/3 phosphorylation. (**g**) Experimental design. Newborn mouse-derived, RepSox-expanded P5 and P20 epidermal keratinocytes were further grown in continuous presence (upper) or absence (middle and lower) of 1 μM RepSox for 24 hrs. Culture of P5 cells stimulated with 1 ng/ml TGF-β for 1 hr prior to lysis (lower) was used as a positive control. (**h**) Expression of total and phosphorylated Smad2/3 as determined by Western blot. Lane numbers correspond to those in (**g**). The full-length blots are included in Supplementary Fig. S11.

Although mouse epidermal cells can proliferate in low Ca^2+^ media, their lifespan is relatively short^16, 24^. As mouse epidermal cells express TGF-β *in vitro* ^28, 29^, we sought to determine whether TGF-β signaling is operative in mouse epidermal cells in primary cultures, thus limiting their proliferation. To address this possibility, we investigated the subcellular localization of Smad2/3 in cytokeratin-positive (CK^+^) mouse primary epidermal cells grown in CnT-PR, one of the best commercially available basal media for low Ca^2+^ epithelial culture. As epidermal cells in newborn mice are more proliferative than those in postnatal mice, we first focused on the analysis of primary cells from newborn mice in this study. We found that a substantial level of Smad2/3 was localized in the nuclei of virtually all CK^+^ newborn mouse epidermal cells, including those of potentially proliferative immature keratinocytes with relatively small nuclear-to-cytoplasmic ratios (Fig. 1a). Notably, however, treatment with RepSox, a cell-permeable and selective inhibitor of the TGF-β type 1 receptor/ALK5, substantially reduced the nuclear localization of Smad2/3, while addition of TGF-β ligands in culture promoted it (Fig. 1a,b). These results indicate that TGF-β signaling is active in mouse primary epidermal cells in a growth-permissive condition and that TGF-β signaling activity can be suppressed by the use of small molecule inhibitors.

Consistent with the inhibition of the nuclear localization of Smad2/3, RepSox treatment enhanced the proliferation of CK^+^ mouse primary epidermal cells while co-existing CK^−^ cells were not responsive to the RepSox treatment (Fig. 1c,d). Accordingly, RepSox treatment robustly increased the CK^+^ epidermal cell number in a dose-dependent manner (Supplementary Fig. S1). Other widely used TGF-β signaling inhibitors with unrelated chemical structures also stimulated the growth of CK^+^ mouse primary epidermal cells in a dose-dependent manner (Supplementary Fig. S1). As the composition of CnT-PR media is not publicly available, we also used a chemically defined alternative, specifically a low Ca^2+^ keratinocyte basal medium, termed SFM. Our data show that RepSox treatment robustly stimulated the growth of CK^+^ mouse epidermal cells in SFM media to an extent similar to that in CnT-PR media (Supplementary Fig. S2). These data indicate that inhibition of TGF-β signaling stimulates the proliferation of mouse primary epidermal cells in basal media.

### Inhibition of TGF-β signaling enables long-term expansion of p63^+^ mouse epidermal progenitor cells

As our data show that TGF-β signaling inhibition stimulates the proliferation of CK^+^ mouse primary epidermal cells but not of CK^−^ cells (Fig. 1d), we next determined whether RepSox treatment enriches p63^+^ epidermal progenitor cells in crude tissue preparations. Although primary cells harvested from newborn mouse skin included 40% to 50% p63-negative (p63^−^) cells at passage 1 (P1) in the absence of RepSox (Figs 1e and S3), addition of RepSox in culture yielded approximately 80% of cells with p63 expression (Fig. 1e). As low Ca^2+^ media preferentially expand epidermal cells^16^, virtually all cells in both cultures with and without RepSox were CK^+^p63^+^ by P2. Notably, however, CK^+^p63^+^ epidermal progenitor cells were maintained beyond P5 only in the presence of RepSox, while the absence of RepSox treatment did not allow any cells to survive beyond P4 (Fig. 1e). These results indicate that inhibition of TGF-β signaling enriches p63^+^ epidermal progenitor cells in crude preparations of mouse epidermis in basal media.

Next, we determined the effect of TGF-β signaling inhibition on long-term proliferation of mouse epidermal progenitor cells that had been expanded by RepSox treatment in CnT-PR media. Notably, p63^+^ mouse epidermal progenitor cells grew at a constant rate at least for 60 days in the presence of RepSox (Fig. 1f). Proliferation of epidermal progenitor cells was RepSox-dependent, as removal of RepSox from the culture resulted in a rapid decline in the growth of epidermal cells (Fig. 1f), accompanied by elevated expression of phosphorylated Smad2/3 in both P5 and P20 epidermal progenitor cells (Fig. 1g,h). Consistent with these findings, gene expression analysis showed that RepSox treatment suppressed known TGF-β target CDK inhibitor genes^30–32^, including *p21* ^*Waf1/Cip1*^, *p16* ^*Ink4a*^, *p19* ^*Arf*^, and *p15* ^*Ink4b*^ (Supplementary Fig. S4). These results indicate that inhibition of TGF-β signaling enables long-term expansion of p63^+^ mouse epidermal progenitor cells by promoting cell cycle progression in basal media.

### Inhibition of TGF-β signaling supports self-renewal of mouse primary epidermal progenitor cells in 3T3-J2 co-culture

Clonogenic culture with 3T3-J2 cells has been used to assess self-renewal capacity of epithelial progenitor cells of many different species^10–12, 33^. However, except for some rare cell types (e.g. hair follicle stem cells)^34^, mouse primary epithelial cells do not form macroscopically visible colonies in serial cultures with 3T3-J2 cells, owing at least in part to their short lifespan *in vitro*. Given that inhibition of TGF-β signaling enables long-term proliferation of mouse epidermal progenitor cells (Fig. 1f), we next sought to determine whether TGF-β signaling inhibition supports their self-renewal in serial 3T3-J2 co-culture.

**Figure 2:**
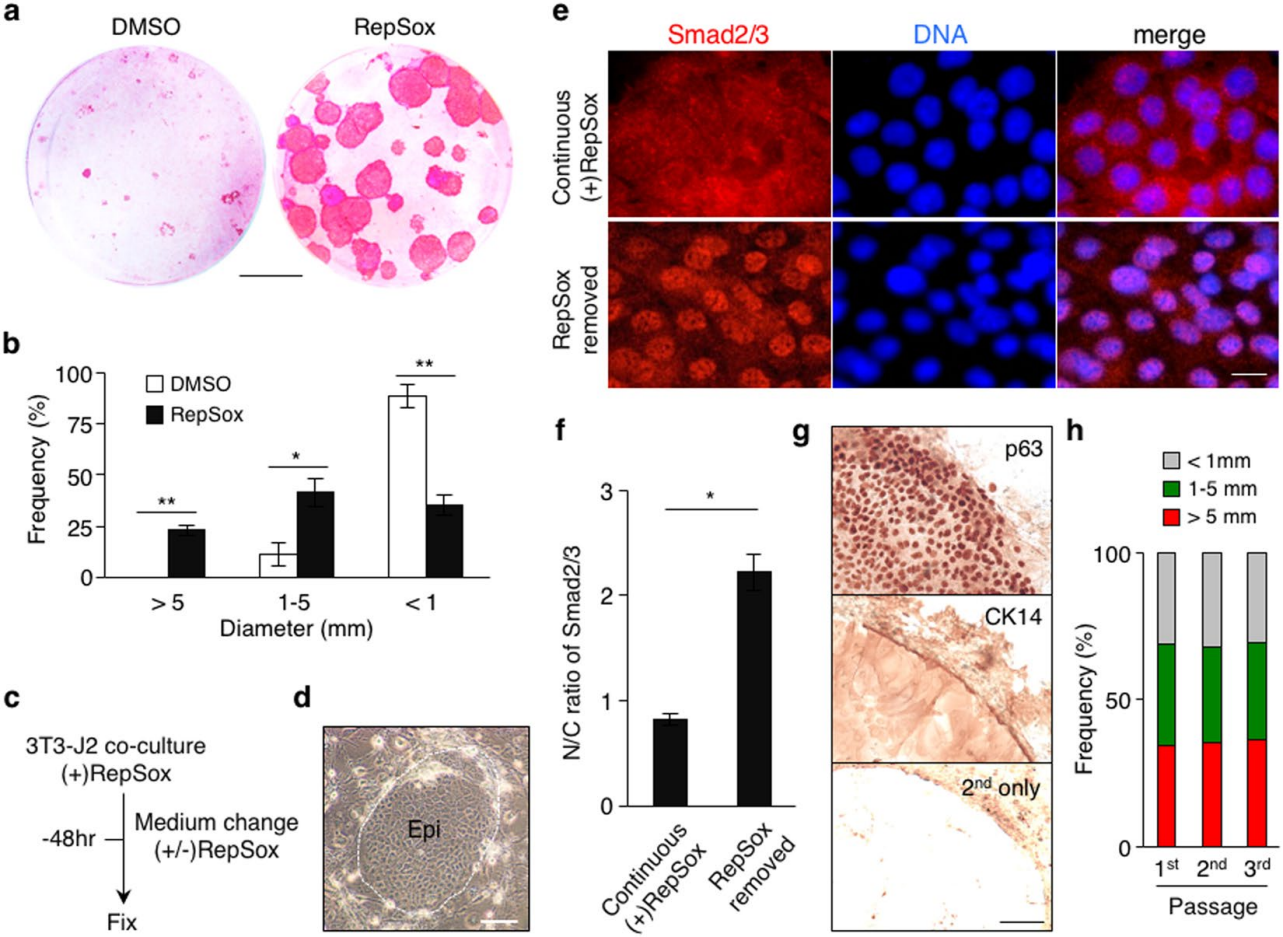
Inhibition of TGF-β signaling supports self-renewal of mouse primary epidermal progenitor cells in serial 3T3-J2 co-culture. (**a**) Rhodamine B staining of newborn mouse-derived, RepSox-expanded P1 epidermal cells grown in 3T3-J2 co-culture in the presence or absence of 1 μM RepSox for 14 days. Bar = 10 mm. (**b**) Distribution of epidermal clone sizes at day 14. Data shown are mean ± s.e.m. (n = 3). *P < 0.05; **P < 0.01. (**c**–**f**) Removal of RepSox associates with nuclear translocation of Smad2/3 in newborn mouse epidermal cells in 3T3-J2 co-culture. (**c**) Experimental scheme. Mouse primary epidermal keratinocytes were grown in 3T3-J2 co-culture in the presence of 1 μM RepSox. Subsequently, the cultures were maintained in the presence or absence of 1 μM RepSox for 48 hrs before fixation for immunohistochemistry. (**d**) A representative image of mouse primary keratinocyte clones in 3T3-J2 co-culture. Note that epidermal cell clones show a cobblestone appearance with a distinct clone border (dotted line). *Epi*, epidermal cell clone. Bar = 50 μm. (**e**) Representative immunofluorescence images of mouse epidermal cells without (upper) or with (lower) removal of RepSox, stained with anti-Smad2/3 antibodies (left panels) and counterstained with Hoechst 33342 (middle panels). Right panels show merged images. Bar = 10 μm. (**f**) Nuclear-to-cytoplasmic (N/C) ratios of Smad2/3 as determined by fluorescence intensity. Data shown are mean ± s.e.m. (n = 10). *P < 0.001. (**g**) Immunohistochemistry of representative epidermal cell clones grown in 3T3-J2 co-culture for 14 days in the presence of 1 μM RepSox, stained with anti-p63 (upper) and anti-CK14 (middle) antibodies. The lower panel represents a control clone stained with the secondary antibodies alone. Data shown are peripheral regions of three different epidermal clones with similar diameter. Bar = 100 μm. (**h**) Distribution of epidermal clone sizes in serial co-culture with 3T3-J2 cells in the presence of 1 μM RepSox.

The RepSox-expanded newborn mouse epidermal cells were transferred onto 3T3-J2 cells at a clonal density and grown for two weeks in the presence or absence of RepSox. Notably, our data show that inclusion of RepSox in culture drastically enhanced the clonal growth of mouse primary epidermal cells (Fig. 2a,b). Removal of RepSox from the co-culture led to an accumulation of Smad2/3 in the nuclei of epidermal cells (Fig. 2c–f), indicating that TGF-β signaling in epidermal cells inversely correlates with their clonal growth. The majority of large clones grown in the presence of RepSox were composed of cytokeratin 14 (CK14)-positive immature epidermal cells with relatively small nuclear-to-cytoplasmic ratios and high p63 levels (Fig. 2g), resembling those in human epidermal stem cell clones termed holoclones^10, 12^. In contrast, the absence of RepSox in 3T3-J2 co-culture only produced smaller colonies and, even in the largest group of clones, the majority of epidermal cells were morphologically differentiated. Although indirect effects by feeder cells under TGF-β signaling inhibition need to be elucidated, these results clearly show that RepSox treatment supports self-renewal of p63^+^ mouse epidermal progenitor cells in 3T3-J2 co-culture.

To determine whether continuous suppression of TGF-β signaling enables long-term assessment of self-renewal of mouse epidermal progenitor cells, we harvested epidermal cells from the RepSox-treated 3T3-J2 co-culture and passaged them on fresh 3T3-J2 cells multiple times at a clonal density with two-week intervals in the presence or absence of RepSox. Our results show that, while in the absence of RepSox epidermal cells no longer formed holoclone-like clones in the subsequent passage, the presence of RepSox continuously produced holoclone-like large colonies at similar rates at least for three generations (Fig. 2h). These results indicate that continuous suppression of TGF-β signaling enables long-term assessment of self-renewal of mouse epidermal progenitor cells in serial 3T3-J2 co-culture.

### Epidermal progenitor cells expanded by TGF-β signaling inhibition are capable of differentiation *in vitro*

**Figure 3:**
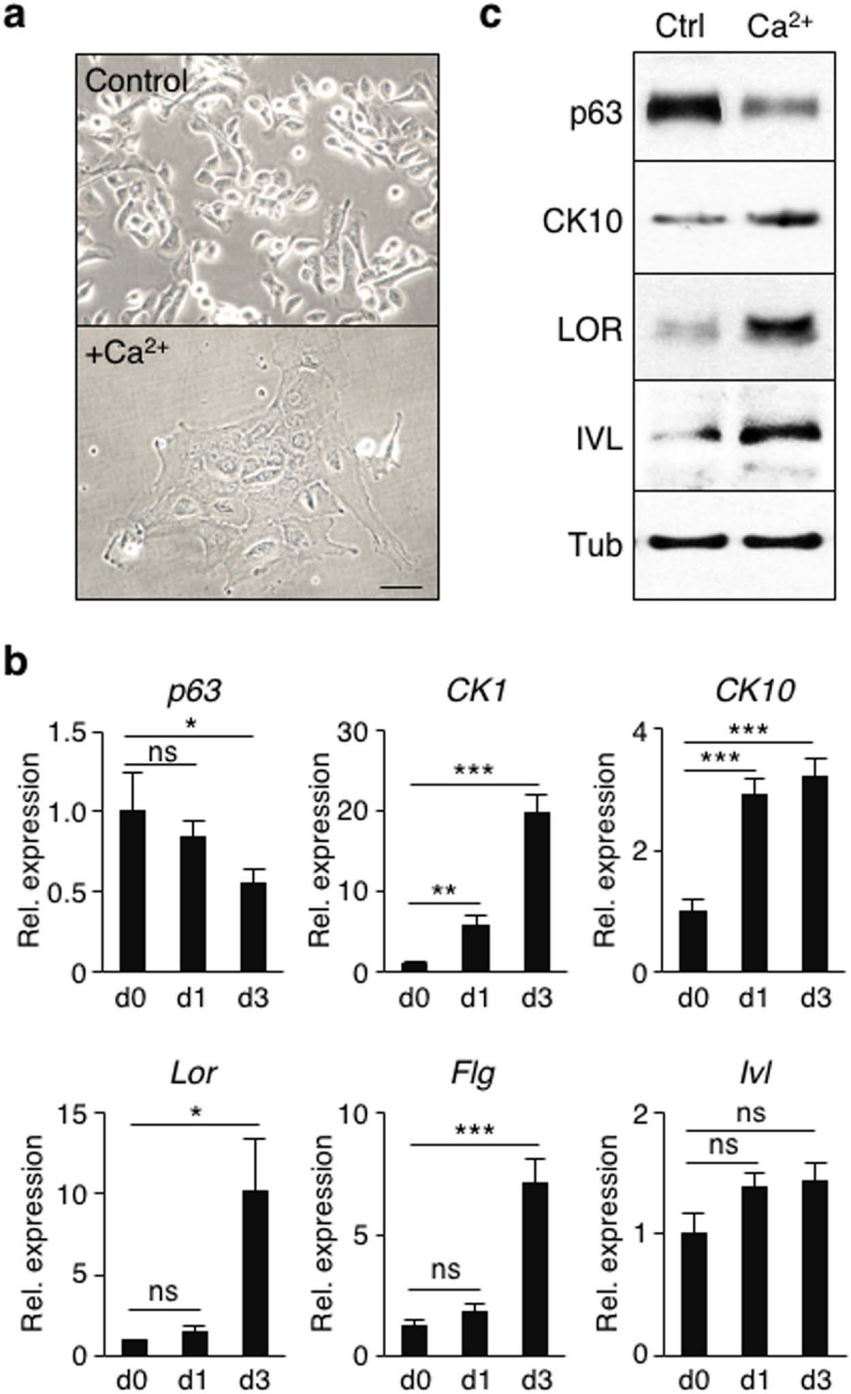
Mouse epidermal progenitor cells expanded by TGF-β inhibition are capable of differentiation in response to Ca^2+^ stimulation *in vitro*. (**a**) Representative images of newborn mouse-derived, RepSox-expanded P18 epidermal cells before (upper) and after (lower) treatment with 0.3 mM Ca^2+^ for 3 days. Bar = 25 μm. (**b**) Quantitative RT-PCR analysis of epidermal cell differentiation marker genes. Newborn mouse-derived, RepSox-expanded P17 epidermal cells were induced to differentiate by treatment with 0.3 mM Ca^2+^ (for *CK1*, *CK10*, *Lor*, *Flg*, and *Ivl*) or 1.3 mM Ca^2+^ (for *p63*) for 0–3 days as indicated. Data shown are normalized to the housekeeping gene *Gapdh* and expressed as mean ± s.e.m. (n = 3). *P < 0.05; **P < 0.01; ***P < 0.005; *ns*, not significant. (**c**) Western blot analysis using antibodies against p63, CK10, loricrin (LOR), and involucrin (IVL). Newborn mouse-derived, RepSox-expanded P17 epidermal cells were induced to differentiate by the treatment with 0.3 mM Ca^2+^ for 0 (Ctrl) and 3 (Ca^2+^) days. Tubulin-α (Tub) was used as a loading control. The full-length blots are included in Supplementary Figure S12.

To determine whether mouse epidermal progenitor cells expanded by TGF-β signaling inhibition are capable of differentiation *in vitro*, we performed Ca^2+^-mediated differentiation^35^ of epidermal progenitor cells that had been expanded by RepSox treatment in primary culture of newborn mouse epidermis. Treatment of the cells with Ca^2+^ for 3 days in the absence of RepSox led to an enlarged and flattened morphology with an increase in nuclear-to-cytoplasmic ratios, suggesting that they are differentiating (Fig. 3a). This is supported by changes in expression of epidermal cell differentiation markers (Fig. 3b,c). For example, treatment with Ca^2+^ decreased p63 expression at both mRNA and protein levels although a higher concentration of Ca^2+^ (1.3 mM) was required for this effect on mRNA levels than protein levels (0.3 mM Ca^2+^). These results are consistent with a previous report showing that p63 is subject to proteasome-mediated degradation during keratinocyte differentiation^36^. Expression of CK1, CK10, and loricrin, early-to-intermediate differentiation markers^37–39^, increased in response to 0.3 mM Ca^2+^ treatment (Fig. 3b,c). Likewise, expression of filaggrin and involucrin, markers for terminal differentiation^40, 41^, also increased by 0.3 mM Ca^2+^ treatment although the transcription of the *Ivl* gene remained relatively unchanged (Fig. 3b,c). These results indicate that mouse epidermal progenitor cells expanded by TGF-β signaling inhibition are capable of differentiation in response to Ca^2+^ stimulation *in vitro*.

### Inhibition of TGF-β signaling enables long-term expansion of p63^+^ epidermal progenitor cells of postnatal mice

**Figure 4:**
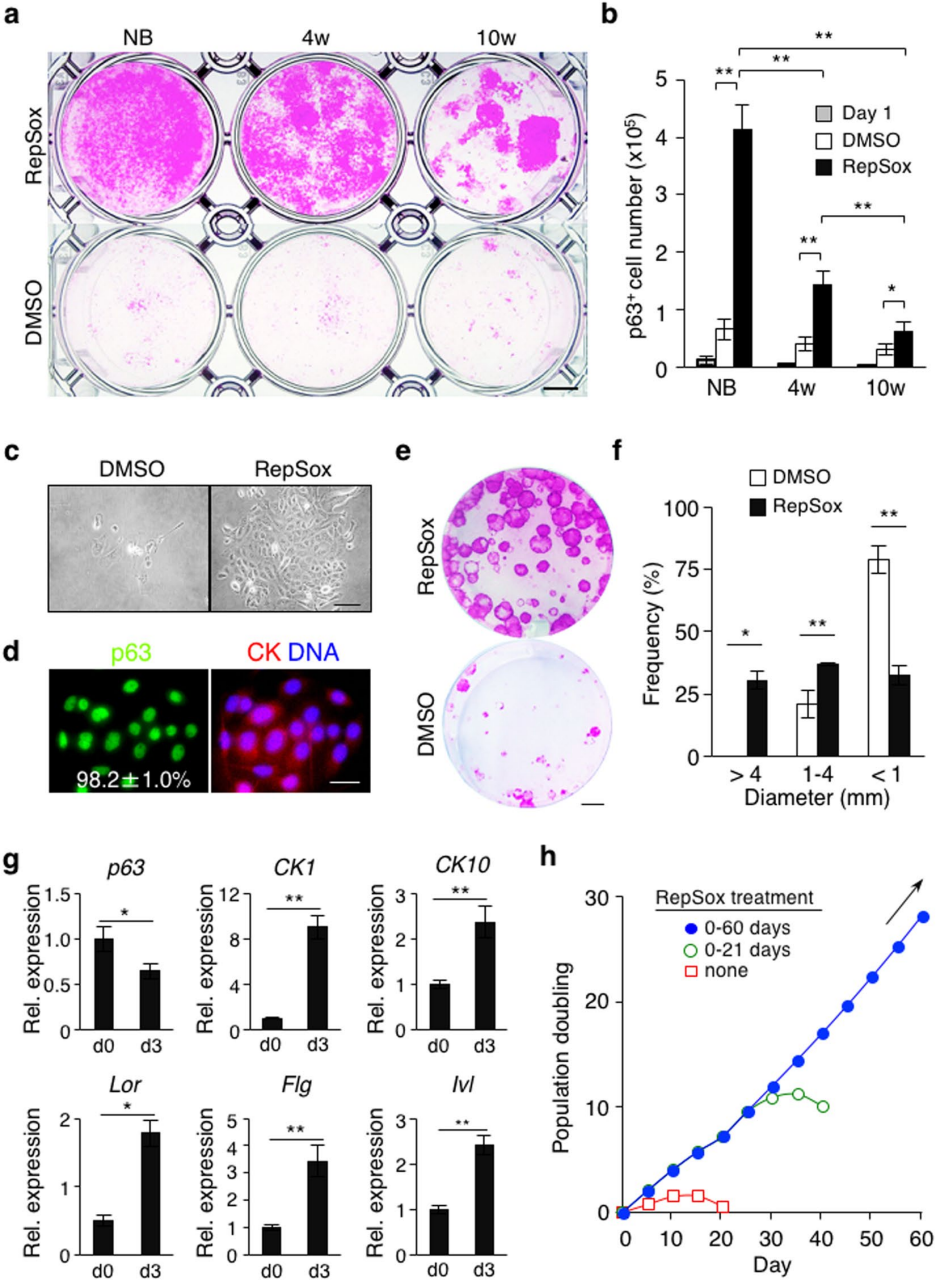
Inhibition of TGF-β signaling supports high proliferative potential of p63^+^ epidermal progenitor cells of postnatal mice. (**a**) Representative images of Rhodamine B staining of crude culture of epidermal cells harvested from newborn (NB), 4-week-old (4w), and 10-week-old (10w) mice and grown in CnT-PR media in the presence (upper) or absence (lower) of 1 μM RepSox for 14 days. One million primary cells were seeded. Data shown are representative of two independent experiments with similar results. Bar = 5 mm. (**b**) Total number of p63^+^ epidermal cells. One million primary cells were seeded and adherent cells were counted at day1 in replicative wells (grey bars) as the majority of the cells remained in suspension. Solid bars and open bars represent total numbers of p63^+^ epidermal cells at day 14 of culture in the subsequent presence of 1 μM RepSox or 0.1% DMSO, respectively (n = 3). *P < 0.05; **P < 0.01. (**c**) Representative images of 4-week-old mouse-derived P1 epidermal cells, grown in the presence (right) or absence (left) of 1 μM RepSox for 6 days. Bar = 50 μm. (**d**) Representative immunofluorescence images of 4-week-old mouse-derived, RepSox-expanded P1 epidermal cells stained with anti-p63 and anti-pan-CK antibodies and counterstained with Hoechst 33342 (DNA). The number shown in the left panel represents percentages of p63^+^ cells per field, expressed as mean ± s.e.m. (n = 4). Bar = 25 μm. (**e**) Representative images of Rhodamine B staining of epidermal clones grown for 14 days in the presence (upper) or absence (lower) of 1 μM RepSox. Four-week-old mouse-derived, RepSox-expanded P2 epidermal cells were used. Bar = 5 mm. (**f**) Distribution of epidermal clone sizes at day 14. Data shown are mean ± s.e.m (n = 3). *P < 0.05; **P < 0.01. (**g**) Quantitative RT-PCR analysis of epidermal cell differentiation marker genes. Four-week-old mouse-derived, RepSox-expanded P7 epidermal cells were induced to differentiate by treatment with 0.3 mM Ca^2+^ (for *CK1*, *CK10*, *Lor*, *Flg*, and *Ivl*) or 1.3 mM Ca^2+^ (for *p63*) for 3 days. Data shown are normalized to the housekeeping gene *Gapdh* and expressed as mean ± s.e.m (n = 3). *P < 0.05; **P < 0.01. (**h**) Population doubling of 4-week-old mouse-derived, RepSox-expanded P2 epidermal cells grown in CnT-PR media in the presence of 1 μM RepSox for 0, 21, and 60 days. Data shown are representative of three independent experiments with similar results. Arrow indicates continuous cell growth.

Our studies thus far utilized newborn mice to demonstrate that inhibition of TGF-β signaling enables long-term expansion of p63^+^ epidermal progenitor cells. To determine whether TGF-β signaling inhibition also expands p63^+^ epidermal progenitor cells of postnatal mice, we first cultivated crude primary cells from newborn, 4-week-old, and 10-week-old mouse epidermis in CnT-PR media (Fig. 4a,b). Our data show that, albeit at a lower level compared to the primary culture of newborn mouse epidermis, TGF-β signaling inhibition produced significant numbers of p63^+^ epidermal cells in cultures of 4-week-old mouse epidermis, and somewhat less in cultures of 10-week-old mouse epidermis (Fig. 4a,b). Similar to newborn mouse epidermis (Fig. 1e), RepSox treatment in crude culture of 4-week-old mouse epidermis yielded over 90% p63^+^ progenitor cells at P1 (Fig. 4c,d). Subsequently, RepSox-expanded P2 epidermal cells showed high clonogenic potential in 3T3-J2 co-culture (Fig. 4e,f) and were capable of differentiation in response to Ca^2+^ stimulation (Fig. 4g). We also show that p63^+^ epidermal progenitor cells derived from 4-week-old mice grew at a constant rate for at least 60 days in a RepSox-dependent manner (Fig. 4h). Together, these results indicate that inhibition of TGF-β signaling in crude primary cell culture of postnatal mouse epidermis selectively expands p63^+^ epidermal progenitor cells and supports their proliferative potential long term.

### Inhibition of TGF-β signaling supports high proliferative potential of p63^+^ epithelial progenitor cells of diverse mouse epithelia

**Figure 5:**
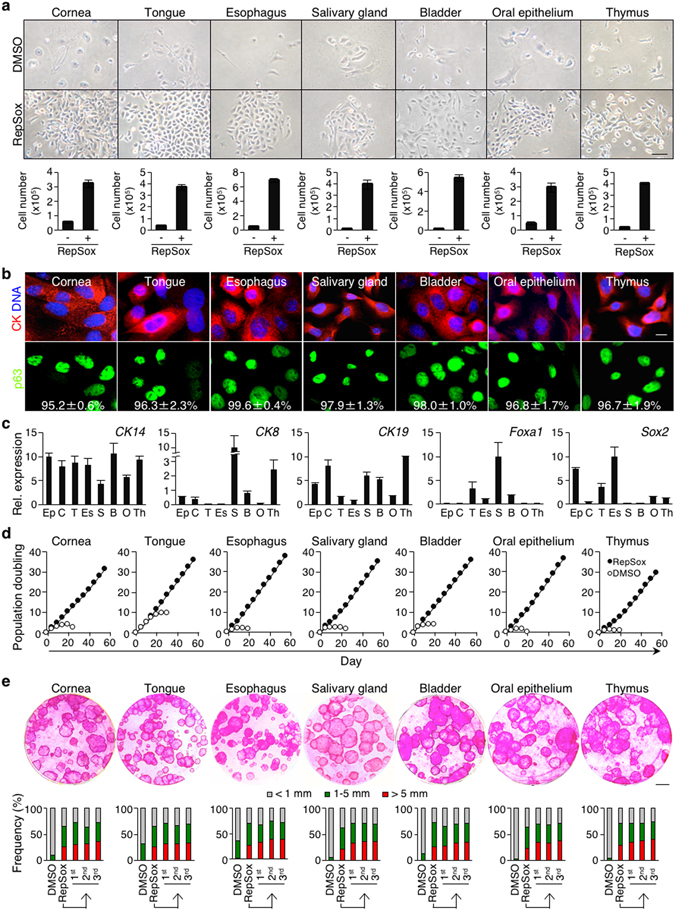
Inhibition of TGF-β signaling enables long-term expansion of p63^+^ epithelial progenitor cells of diverse mouse epithelia. (**a**) Representative images of primary cells of various newborn mouse epithelia, grown in the presence (lower) or absence (upper) of 1 μM RepSox for 6 days. Bar = 50 μm. Bar graphs below indicate numbers of primary cells at day 7 of culture. CnT-PR-expanded CK^+^ mouse primary epithelial cells (2 × 10^4^) were seeded. Data shown are mean ± s.e.m. (n = 3). (**b**) Representative immunofluorescence images of newborn mouse-derived, RepSox-expanded P2 epithelial cells stained with anti-p63 and anti-pan-CK antibodies and counterstained with Hoechst 33342 (DNA). Numbers shown in lower panels represent percentages of p63^+^ cells per field, expressed as mean ± s.e.m. (n ≥ 4). Bar = 10 μm. (**c**) Quantitative RT-PCR analysis of epithelial progenitor cell-associated genes using newborn mouse-derived, RepSox-expanded P9 (thymus and salivary gland), P10 (epidermis), and P11 (cornea, oral epithelium, tongue, esophagus and bladder) cells. For each gene, the highest expression was set to 10. Data shown are normalized to the housekeeping gene *Rps18* and expressed as mean ± s.e.m. (n = 3). *Ep*, epidermis; *C*, cornea; *T*, tongue; *Es*, esophagus; *S*, salivary gland; *B*, bladder; *O*, oral epithelium; and *Th*, thymus. (**d**) Population doubling of newborn mouse-derived, RepSox-expanded P2 epithelial cells grown in CnT-PR media in the presence (solid) or absence (open) of 1 μM RepSox. (**e**) 3T3-J2 co-culture. Representative images of Rhodamine B staining of epithelial clones grown for 14 days in the presence of 1 μM RepSox. Newborn mouse-derived, RepSox-expanded P1 epithelial cells were used. Bar = 5 mm. Data shown below indicate the distribution of epithelial clone sizes during serial 3T3-J2 co-cultures. Epithelial cells harvested from RepSox-treated primary 3T3-J2 co-cultures (lane 2) were serially cultivated with two-week intervals with fresh 3T3-J2 cells in the presence of 1 μM RepSox (lanes 3–5). *DMSO*, primary cultures without RepSox (lane 1).

As p63 is expressed in the stem cell compartment of diverse epithelia in mice^42^, we next determined whether TGF-β signaling inhibition would also enrich and expand p63^+^ progenitor cells of epithelia in mouse tissues other than epidermis. To begin with, we prepared crude primary cells of various newborn mouse epithelia that contain p63^+^ progenitor cells, including the cornea, tongue, esophagus, salivary gland, bladder, oral epithelium, and thymus (Supplementary Fig. S5) and placed them in CnT-PR media in the presence or absence of RepSox. To validate the feasibility of TGF-β signaling inhibition in primary cultures of postnatal mouse epithelia, we also included some representative 4-week-old mouse epithelia, including the esophagus, salivary gland, bladder, and thymus. Our data show that RepSox treatment robustly enhanced the growth of primary cells of all newborn and 4-week-old mouse epithelia we examined (Figs 5a and S6a). In contrast, RepSox treatment in CnT-PR culture failed to stimulate the growth of primary cells of p63-independent epithelia, such as the small intestine and colon^14, 15^, (Supplementary Fig. S7). Although crude preparations of primary cultures of all p63-dependent epithelia contained non-epithelial cells and terminally differentiated p63^−^ epithelial cells, just as in epidermis, >90% of cells from all epithelial cultures showed high p63 expression by P1 to P2 (Figs 5b and S6b), and virtually all growing cells were p63^+^ thereafter in all cultures derived from both newborn and 4-week-old mice. These results indicate that inhibition of TGF-β signaling in CnT-PR media selectively expands p63^+^ epithelial progenitor cells in crude tissue preparations of diverse mouse epithelia.

To determine whether epithelial progenitor cells express markers for the corresponding progenitor cells in each epithelium after prolonged exposure to TGF-β inhibition, we examined relative expression of five representative, well-characterized genes, including *CK14*, *CK8*, *CK19*, *Foxa1*, and *Sox2*, using RepSox-expanded P9 to P11 newborn mouse epithelial progenitor cells (Fig. 5c). Consistent with uniformly high expression of p63, *CK14*, a common basal epithelial cell marker^43^, was expressed in all progenitor cells to a similar extent (Fig. 5c). In contrast, expression of tissue-specific CK markers^44–49^, *CK8* and *CK19*, was relatively higher in progenitor cells of the salivary gland, thymus, bladder, and cornea than in the other progenitor cells (Fig. 5c). Expression of the transcription factor *Foxa1* ^50^ was substantially higher in the progenitor cells of the salivary gland, tongue, bladder, and esophagus than in the other progenitor cells, while *Sox2* transcription factor^51, 52^ was expressed at considerably higher levels in the progenitor cells of the esophagus, epidermis, tongue, oral epithelium, and thymus than in the other progenitor populations (Fig. 5c). These results are consistent with previous reports on marker expression in tissue-specific progenitor cells^43–52^, suggesting that the gene expression signature in each progenitor population is preserved after prolonged exposure to TGF-β signaling inhibition.

Next, we determined whether inhibition of TGF-β signaling supports long-term proliferation and self-renewal of epithelial progenitor cells. Our data show that RepSox treatment robustly stimulated the expansion of all newborn and 4-week-old mouse epithelial progenitor cells long term, for at least 60 days when the experiment was terminated (Figs 5d and S6c). Our data also show that RepSox-expanded progenitor cells of all newborn and 4-week-old mouse epithelia produced holoclone-like large clones in 3T3-J2 co-cultures (Figs 5e and S6d), and in the case of newborn mouse epithelial progenitor cells, they all produced large clones at similar rates in three consecutive subcultures in the presence of RepSox (Fig. S5e, lower panels). The absence of RepSox in subculture produced very small or abortive clones that did not survive in the subsequent passage. These results indicate that inhibition of TGF-β signaling supports long-term proliferation and self-renewal of epithelial progenitor cells of diverse mouse epithelia.

**Figure 6:**
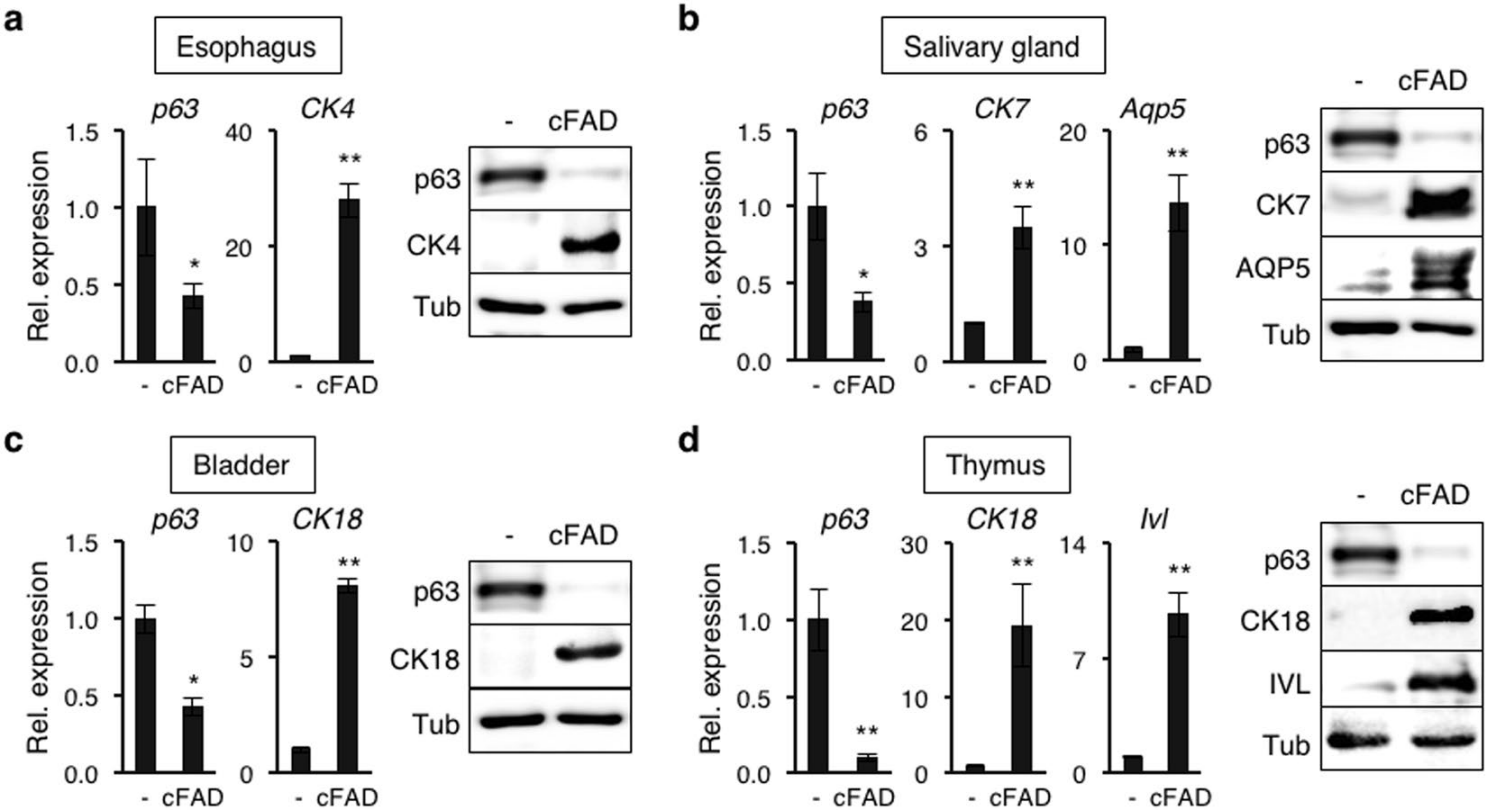
Mouse epithelial progenitor cells expanded by TGF-β inhibition are capable of differentiation *in vitro*. (**a**–**d**) Newborn mouse-derived epithelial progenitor cells expanded by RepSox treatment were grown in CnT-PR media in the presence of 1 μM RepSox to sub-confluence, followed by cultivation in cFAD for 7 days in the absence of RepSox. Epithelial progenitor cells used were derived from the (**a**) esophagus, (**b**) salivary gland, (**c**) bladder, and (**d**) thymus. *Bar graphs on the left*, quantitative RT-PCR analysis of epithelial cell differentiation marker genes, *p63*, *CK4*, *CK7*, *CK18*, *Ivl*, and *Aqp5*. Data shown are normalized to the housekeeping gene *Gapdh* and expressed as mean ± s.e.m. (n = 3). *P < 0.05; **P < 0.01. *Right*, Western blot analysis with antibodies against p63, CK4, CK7, CK18, aquaporin 5 (AQP5), and involucrin (IVL). Tubulin-α (Tub) was used as a loading control. The full-length blots are included in Supplementary Figure S13.

Finally, we determined whether mouse epithelial progenitor cells expanded by TGF-β signaling inhibition are capable of differentiation *in vitro* using the esophagus, salivary gland, bladder, and thymus of both newborn and 4-week-old mice. To induce differentiation of epithelial progenitor cells, RepSox-expanded p63^+^ progenitor cells derived from each epithelium were cultivated in cFAD media^10^ in the absence of RepSox. Although cFAD has been developed for stimulating the growth of human epidermal keratinocytes in 3T3-J2 feeder cell co-culture, it allows differentiation of mouse epidermal progenitor cells in monolayer culture without 3T3-J2 cell support (Supplementary Fig. S8). Similar to epidermal progenitor cells, our data show that all epithelial progenitor cells of both newborn and 4-week-old mice tested decreased *p63* expression in culture with cFAD as determined by qPCR (Figs 6a–d and S6e). Decrease in p63 protein levels was verified by Western blot using newborn mouse epithelial progenitor cells (Fig. 6a–d). We then analyzed expression of epithelial cell differentiation markers that are relatively well characterized and unique to each epithelial cell type. Our data show that expression of cytokeratin 4 (*CK4*), a marker of suprabasal cells in the esophagus^53^, increased by 28-fold and 10-fold in newborn and 4-week-old mouse epithelial progenitor cells, respectively, in response to cFAD treatment (Figs 6a and S6e). Expression of cytokeratin 7 (*CK7*) and aquaporin 5 (*Aqp5*), lineage-specific markers of ductal epithelia and acinar epithelia in the salivary glands, respectively^54^, significantly increased in progenitor cells derived from both newborn and 4-week-old mice in response to cFAD treatment (Figs 6b and S6e). Likewise, expression of cytokeratin 18 (*CK18*), a marker of the superficial layer of the bladder^55^, substantially increased in both newborn and 4-week-old mouse-derived epithelial progenitor cells in response to cFAD treatment (Figs 6c and S6e). Lastly, expression of *CK18* and *Ivl*, markers of cortical epithelia and medullary epithelia of the thymus, respectively^56, 57^, increased significantly in both newborn and 4-week-old mouse epithelial progenitor cells upon cultivation in cFAD (Figs 6d and S6e). Increase of all differentiation markers at the protein levels was verified by Western blot using newborn mouse epithelial progenitor cells (Fig. 6a–d). Although the mechanisms of how cFAD induces differentiation of each epithelial cell type need to be determined, these results indicate that p63-dependent mouse epithelial progenitor cells expanded by TGF-β signaling inhibition are capable of differentiation *in vitro*.

## Discussion

In this report, we describe a simple and effective method of expanding p63^+^ epithelial progenitor cells from crude tissue preparations of diverse mouse epithelia using a single small molecule inhibitor of TGF-β signaling in a low Ca^2+^, feeder-free condition. Although dedifferentiation of terminally differentiated p63^−^ epithelial cells into p63^+^ progenitor cells and/or transdifferentiation of p63^−^ non-epithelial cells into a p63^+^ epithelial lineage could contribute to the observed increase of p63^+^ epithelial progenitor cell numbers, our data described below suggest that these two possibilities are unlikely. First, terminally differentiated mouse keratinocytes induced by Ca^2+^ stimuli remained non-proliferative upon exposure to TGF-β signaling inhibition (Supplementary Fig. S9). Second, inhibition of TGF-β signaling did not allow any cells from tissues supported by p63-independent progenitor cells (i.e. small intestine and colon) to expand in CnT-PR media (Supplementary Fig. S7), likely due to a lack of growth supporting factors for these epithelial cell types^58, 59^. Thus, we conclude that inhibition of TGF-β signaling in CnT-PR or other low Ca^2+^ basal media selectively expands p63^+^ epithelial progenitor cells without involving dedifferentiation of terminally differentiated p63^−^ epithelial cells and/or transdifferentiation of p63^−^ non-epithelial cells. This is particularly important as our methodology can significantly reduce the labor and cost associated with progenitor cell pre-purification steps, while efficiently expanding highly enriched populations of p63^+^ epithelial progenitor cells with high proliferative capacity.

Our data show that the numbers of p63^+^ epidermal progenitor cells expanded by RepSox treatment in culture declined in an age-dependent manner (Fig. 4b, solid bars). As cultures of newborn, 4-week-old, and 10-week-old mouse epidermal cells produced similar numbers of p63^+^ progenitor cells without RepSox treatment (Fig. 4b, open bars), these results suggest that the relative insensitivity to TGF-β signaling inhibition, rather than a decrease in proliferative cell numbers, was responsible for the age-associated decline in the expansion of p63^+^ epidermal progenitor cells. Although less profound, similar age-associated decrease in RepSox-mediated expansion of p63^+^ progenitor cells was observed in the other epithelial tissues, such as the esophagus, salivary gland, bladder, and thymus (Figs 5d and S6c). Despite intensive studies, however, the mechanisms that regulate age-associated alterations in quantity and/or quality of progenitor cells are controversial^60^, likely due to the involvement of complex signaling acting in these processes in both cell-autonomous and extrinsic manner. In this regard, it is important to note that our present work will provide a unique opportunity to assess age-dependent alterations in TGF-β signaling that may control versatile cell-intrinsic properties^61^ of a diverse array of p63^+^ epithelial progenitor cells.

Recently, it has been shown that several different mouse epithelial progenitor cells can be expanded from p63-dependent epithelia, such as the skin, prostate, mammary gland, and thymus, in 3T3-J2 co-culture in the presence of Rho-associated protein kinase (Rock) inhibitors^20, 22, 62–64^. Although the molecular mechanisms underlying Rock inhibition-mediated expansion of epithelial progenitor cells are unclear, TGF-β signaling has been shown to regulate the Rho signaling pathway in normal mouse mammary gland epithelial cells^65^. In addition, TGF-β signaling phosphorylates p63, which in turn leads to decreased proliferative capacity of epithelial progenitor cells^66, 67^. Elucidating potential mechanisms by which TGF-β and Rho signaling pathways cooperate with p63 may help to better understand the molecular mechanisms controlling the proliferative potential of epithelial progenitor cells. Nonetheless, the development of feeder-free culture of p63^+^ mouse primary epithelial progenitor cells described in this study will allow us to better perform many downstream applications of epithelial analysis, in which high numbers of p63^+^ epithelial progenitor cells are required. Some examples of such applications would include gene expression studies, molecular and cellular analyses *in vitro*, and manipulation of stem cells *ex vivo*, followed by their transplantation and characterization *in vivo*.

Smad-dependent growth control and differentiation are mediated by both TGF-β and bone morphogenetic protein (BMP) signaling pathways in a context-dependent manner^25^. Indeed, dual inhibition of both pathways has been shown to synergistically promote neural conversion of embryonic stem (ES) cells and induced pluripotent stem (iPS) cells^68, 69^. A recent report by Mou *et al*. has described an analogous approach in which dual inhibition of TGF-β and BMP signaling, in addition to Rock inhibition, can expand mouse airway epithelial progenitor cells^23^. However, it is not clear if inhibition of BMP signaling is required for promoting proliferation of other mouse epithelial progenitor cells. Indeed, our data show that inhibition of BMP signaling did not improve the enhanced proliferation of mouse primary epidermal progenitor cells mediated by TGF-β signaling inhibition (Supplementary Figs S1 and S10). Nevertheless, we showed that inhibition of TGF-β signaling selectively expands diverse p63^+^ mouse epithelial progenitor cells and increases their cellularity by 10^9^-fold (equivalent to PD 30) to 10^12^-fold (PD 40) within 60 days (Figs 1f, 4h and 5d and S6c). Mou *et al*. achieved similar results by dual TGF-β/BMP inhibition in mouse airway epithelial progenitor cells^23^. These data strongly suggest that inhibition of TGF-β signaling alone is sufficient for the expansion of p63^+^ epithelial progenitor cells of many different mouse epithelia.

In conclusion, we have shown that inhibition of TGF-β signaling in basal media enables the expansion of p63^+^ epithelial progenitor cells without the need of progenitor cell-purification steps or a feeder cell layer. We anticipate that this simple and effective approach to enrich and propagate p63^+^ primary epithelial progenitor cells will facilitate the use of normal as well as genetically engineered mouse models for downstream analyses of diverse p63-dependent epithelia.

## Methods

### Mice

All experimental procedures were carried out in accordance with protocols approved by the Institutional Animal Care and Use Committees (IACUC) at the University of Pennsylvania and Boston University. Mice used in this study were on a C57BL/6 background (Jackson Laboratories, Bar Harbor, ME; Charles River, Wilmington, MA). The day of the vaginal plug was designated embryonic day 0.5 (E0.5).

### Tissue isolation and cell culture

All tissues harvested were sterilized in Isojin (Meiji Seika Pharma Co., Ltd., Tokyo, Japan), followed by three washes in phosphate buffered saline (PBS). Primary cells of mouse epidermis were prepared as described^16, 70^ with some modifications. Briefly, newborn mouse skin was harvested from E19.0 to postnatal day 1 mice and incubated in 0.25% trypsin for 2 hrs at room temperature (RT), followed by collection of epidermis using forceps and incubation in 0.25% trypsin for an additional 5 min at RT. Postnatal mouse skin was harvested from 4-week-old and 10-week-old mice and processed as described above. Newborn mouse tissues including the salivary gland, thymus, esophagus, colon, small intestine, and bladder were harvested from E19.0 to postnatal day1 mice, minced into small pieces by scissors, and incubated in 0.25% trypsin for 30 min at 37 °C with vigorous pipetting every 10 min. To isolate primary cells from corneal, lingual, and oral epithelia of newborn mice, eyeballs^71^, tongue^72^, and palatal shelves^73^, respectively, were harvested from E19.0 to postnatal day 1 mice and incubated in 0.25% trypsin for 2 hrs at RT. Loosened epithelia were removed using forceps and incubated in 0.25% trypsin for an additional 5 min at 37 °C. Postnatal mouse tissues including the salivary gland, thymus, esophagus, and bladder were harvested from 4-week-old mice and minced into small pieces by scissors. Subsequently, the salivary gland and thymus were incubated in DMEM containing a mixture of Dispase II (0.5 mg/ml, Roche Diagnostics, Indianapolis, IN), Collagenase (0.5 mg/ml, Worthington, Lakewood, NJ), DNase (0.01 mg/ml, Worthington), and 0.125% trypsin for 20 min at 37 °C with vigorous pipetting every 10 min. The esophagus and bladder were incubated in DMEM containing Dispase II (1 mg/ml), Collagenase (1 mg/ml), and DNase (0.02 mg/ml) for 40 min at 37 °C with vigorous pipetting every 20 min. Dissociated cells from all newborn and postnatal mouse tissues were neutralized with fetal bovine serum (FBS, Hyclone, Logan, UT) and filtered through a 70 μm cell strainer. After centrifugation at 1,500 rpm (400 g) for 5 min, the cells were washed twice in PBS and suspended in CnT-PR epithelial culture basal media (Cellntec, Bern, Switzerland). In some experiments, crude primary cells were plated at a high density and serially passaged 2–3 times in CnT-PR media to deplete all non-epithelial cells. By passage 3, virtually all growing cells in CnT-PR media were cytokeratin (CK)-positive epithelial cells as determined by immunohistochemistry. Other basal media used in this study include Keratinocyte-SFM basal media with growth supplement (Invitrogen). The cells were grown in the presence or absence of 1 ng/ml TGF-β2 (Invitrogen, Carlsbad, CA) or 0.1–10 μM inhibitors (RepSox, A83-01, LY364947, SB525334, SB431542, and DMH-1) (all from Cayman Chemical, Ann Arbor, MI) as indicated. All primary cell cultures were performed in a humidified chamber at 37 °C with 5% CO_2_.

To assess cell numbers, the cells were incubated in 0.25% trypsin for 5–10 min, neutralized with 10% FBS in DMEM, and washed in PBS, followed by cell counting in a glass hemocytometer chamber (Hausser Scientific, Horsham, PA) under a CKX41 Inverted Microscope (Olympus, Center Valley, PA) with a 10X objective. To measure population doubling (PD), the cells were seeded at a density of 2 × 10^4^ cells per well in 12-well plates, grown in CnT-PR media in the presence or absence of 1 μM RepSox, and serially passaged every 5 days. PD was calculated as *PD* = *3*.*32* (*log* [*cell number yielded/cell number inoculated*]).

To induce differentiation of epidermal keratinocytes, sub-confluent cells in 12-well plates were washed twice in PBS and cultivated in CnT-PR media containing 0.3 or 1.3 mM CaCl_2_ for 1–5 days as indicated. To induce differentiation of other epithelial progenitor cells of the esophagus, salivary gland, bladder, and thymus, RepSox (1 μM)-expanded cells were grown to sub-confluence in CnT-PR media in 12-well plates, followed by cultivation in cFAD media for 7 days in the absence of RepSox. In both differentiation assays, culture media were replaced every 24 to 48 hrs and cells were harvested at the time points indicated for subsequent analyses.

### 3T3-J2 co-culture

Clonogenic culture with 3T3-J2 cells was performed as described^10^. Briefly, mouse epithelial cells were seeded at a clonal density on lethally γ-irradiated 3T3-J2 cells (gift from H. Green, Harvard Medical School) in complete FAD (cFAD) media and grown in the presence or absence of 1 μM RepSox in a humidified chamber at 37 °C with 5% CO_2_. To perform serial co-culture with 3T3-J2 cells, epithelial cells were harvested from the initial 3T3-J2 co-cultures by trypsinization in 0.25% trypsin for 5–10 min and equal numbers of epithelial cells were serially cultured with γ-irradiated fresh 3T3-J2 cells with two-week intervals. To visualize epithelial colonies, culture plates were fixed in 10% buffered formalin for 10 min and stained with 1% Rhodamine B (Sigma-Aldrich, St. Louis, MO). To determine clone sizes, the whole plates were photographed with a Nikon digital camera Coolpix S10 (Nikon, Melville, NY) and the diameter of each clone was measured with the aid of Adobe Photoshop (Adobe, San Jose, CA) software.

### Immunohistochemistry and Immunofluorescence

Immunofluorescence and immunohistochemistry were performed as described^67^. Briefly, cultured cells were fixed in 10% buffered formalin for 10 min and permeabilized in PBS/0.1% Triton X-100 for 5 min, followed by blocking with 10% FBS for 30 min and incubation with primary antibodies overnight at 4 °C. After three washes in PBS containing 0.1% Tween-20 (PBST), the cells were incubated with either fluorescence-labeled secondary antibodies for 1 hr followed by counterstaining with 0.1 μg/ml Hoechst 33342 (Invitrogen) or HRP-conjugated secondary antibodies followed by detection of the antigens using a DAB reagent kit (KPL, Gaithersburg, MD).

Mouse tissues were fixed in 10% buffered formalin overnight at 4 °C. Paraffin-embedded sections were cut into 6 μm sections and antigen retrieval was performed by incubating the slides in 0.01 M citric acid buffer (pH6.0) at 95 °C for 20 min. Sections were then blocked with 10% FBS at RT for 30 min and incubated with primary antibodies overnight at 4 °C. After three washes in PBST, the slides were incubated with HRP-conjugated secondary antibodies and the antigens were detected using a DAB reagent kit (KPL).

### BrdU labeling and detection

A short-pulse with 5-bromo-2′-deoxyuridine (BrdU) was performed as described^9^. Briefly, cultured cells were incubated for 30 min with 10 μM BrdU (Roche Diagnostics), fixed in 10% buffered formalin at RT for 10 min, and permeabilized in PBS/0.1% Triton X-100 for 5 min, followed by denaturation in 2 N HCl for 15 min at 37 °C. After blocking with 10% FBS, the cells were incubated with mouse anti-pan-CK and rat anti-BrdU antibodies, and then stained with pre-adsorbed secondary antibodies. The frequency of CK^+^BrdU^+^ cell populations was determined using a Nikon Eclipse 80i microscope and NIS Elements software (Nikon).

### Western blot

Cells were lysed in RIPA buffer containing 50 mM Tris-HCl (pH6.8), 5% 2-mercaptoethanol, 2% sodium dodecyl sulfate, 10% glycerol and 1 mM phenylmethanesulfonyl fluoride (all from Sigma-Aldrich). Equal amounts of proteins were separated by 8% SDS-PAGE and transferred to Immobilon-P membranes (Millipore, Billerica, MA). Blots were developed using the ECL detection reagent (GE Healthcare Bio-Sciences, Pittsburgh, PA).

### Antibodies

Primary antibodies used in this study were mouse anti-p63 (4A4, Santa Cruz Biotechnology, Santa Cruz, CA, sc-8431, 1:1,000 IF/IHC/WB), rabbit anti-p63 (H137, Santa Cruz Biotechnology, sc-8343, 1:100 IF), mouse anti-pan-cytokeratin (CK) (AE1/AE3, Thermo Fisher Scientific, Waltham, MA, MS-343, 1:100 IF), mouse anti-CK4 (6B10, Santa Cruz Biotechnology, sc-52321, 1:100 WB), mouse anti-CK7 (RCK105, Santa Cruz Biotechnology, sc-23876, 1:500 WB), mouse anti-CK10 (RSKE60, Abcam, Cambridge, MA, ab9025, 1:1,000 WB), mouse anti-CK14 (LL001, Santa Cruz Biotechnology, sc-53253, 1:100 IHC), mouse anti-CK18 (RGE53, Santa Cruz Biotechnology, sc-32329, 1:200 WB), rabbit anti-loricrin (BioLegend, San Diego, CA, PRB-145P, 1:4,000 WB), rabbit anti-involucrin (M-116, Santa Cruz Biotechnology, sc-28558, 1:2,000 IF and 1:100 WB), mouse anti-aquaporin 5 (D-7, Santa Cruz Biotechnology, sc-514022, 1:50 WB), mouse anti-tubulin-α (12G10, Developmental Studies Hybridoma Bank, University of Iowa, 1:500 WB), rat anti-BrdU (ICR1, Abcam, ab6326, 1:100 IF), rabbit anti-phosphorylated Smad2/3 (D27F4, Cell Signaling Technology, Danvers, MA, #8628, 1:1,000 WB), and rabbit anti-Smad2/3 (D7G7, Cell Signaling Technology, #8685, 1:1,000 IF/WB).

Secondary antibodies used for immunofluorescence were Alexa 488-goat anti-mouse IgG, Alexa 488-goat anti-rabbit IgG, Alexa 594-goat anti-mouse IgG, Alexa 594-goat anti-rabbit IgG, (Molecular Probes, Grand Island, NY, 1:1,000 IF), pre-adsorbed DyLight488-goat anti-rat IgG, and pre-adsorbed DyLight594-goat anti-mouse IgG (Abcam, 1:1,000 IF). Secondary antibodies used for immunohistochemistry and Western blot were horseradish peroxidase (HRP)-conjugated goat anti-mouse IgG (KPL, 1:250 IHC and 1:5,000 WB), and HRP-conjugated goat anti-rabbit IgG (Cell Signaling Technology, 1:500 WB).

### RNA isolation and quantitative RT-PCR

Total RNA was isolated using Trizol reagent according to the manufacturer’s instructions (Invitrogen) and 1 μg total RNA was reverse-transcribed using the ProtoScript M-MuLV First Strand cDNA Synthesis Kit (New England Biolabs, Ipswich, MA). Quantitative PCR was performed using the SYBR Green PCR Master Mix on an ABI 7900 HT or an ABI StepOnePlus machine according to the manufacturer’s instructions (Invitrogen). Relative expression of each gene was determined using the ∆∆Ct method and normalized to the housekeeping gene *Gapdh* or *Rps18* as indicated. Primer sequences used in this study are listed in Supplementary Table S1.

### Image analysis

Imaging was done on a Nikon Eclipse 80i microscope with a Nikon DS-Qi1Mc digital camera (Nikon) or a BZ-X710 microscope (Keyence Corp., Osaka, Japan) and analyzed with ImageJ (NIH, Bethesda, MD) and Adobe Photoshop software. To determine the nuclear-to-cytoplasmic ratios, signal intensities of Smad2/3 staining in the nucleus (N^Smad2/3^) and cytoplasm (C^Smad2/3^) of individual cells were measured using ImageJ and applied to the equation *Ratio* = (*N* ^*Smad2*/*3*^ − *BG*)/(*C* ^*Smad2*/*3*^ − *BG*), where BG indicates a background signal. To determine BrdU^+^, p63^+^, and CK^+^ cell numbers, randomly selected areas were photographed (n = 3–5 fields) and the captured immunofluorescence images were processed for manual counting of positive cells using Adobe Photoshop software. Counterstaining with Hoechst 33342 was used to determine the total cell numbers.

### Statistical analysis

Values are reported as mean ± standard error of the mean (s.e.m.). Student’s *t*-tests were performed where P < 0.05 was considered statistically significant.

### Data Availability

All data generated or analyzed during this study are included in this published article and its Supplementary Information files.

## Electronic supplementary material

Supplementary Information
