# Supplementary material for: Inhibition of TGF-β signaling supports high proliferative potential of diverse p63+ mouse epithelial progenitor cells in vitro
Source: Sci Rep. 2017 Jul 20;7:6089. doi: 10.1038/s41598-017-06470-y (PMC5519764; doi:10.1038/s41598-017-06470-y)
Supplement: Supplementary file 1 — Supplementary Information [file 41598_2017_6470_MOESM1_ESM.pdf]

Supplementary Information

Inhibition of TGF-β signaling supports high proliferative potential of diverse p63^{+} mouse epithelial progenitor cells *in vitro*

Daisuke Suzuki, Filipa Pinto, and Makoto Senoo

Supplementary Figure S1-S13
Figure Legend for Supplementary Figure S1-S13
Supplementary Table S1

Figure S1

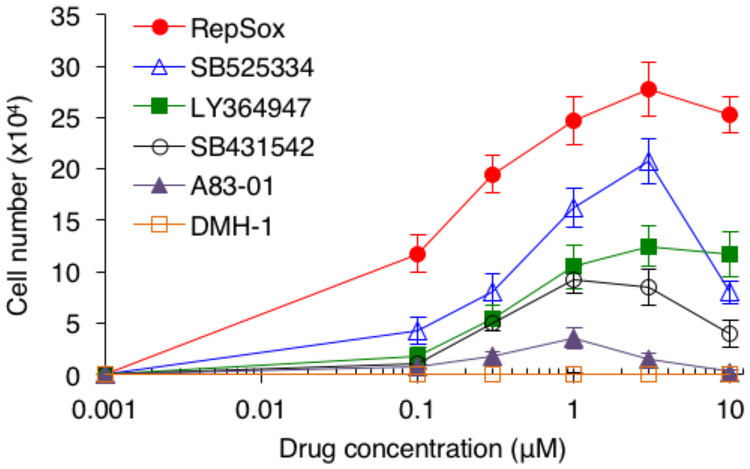
RepSox
SB525334
LY364947
SB431542
A83-01
DMH-1
Cell number (x10⁴)
35
30
25
20
15
10
5
0
0.001
0.01
0.1
1
10
Drug concentration (μM)

Figure S2

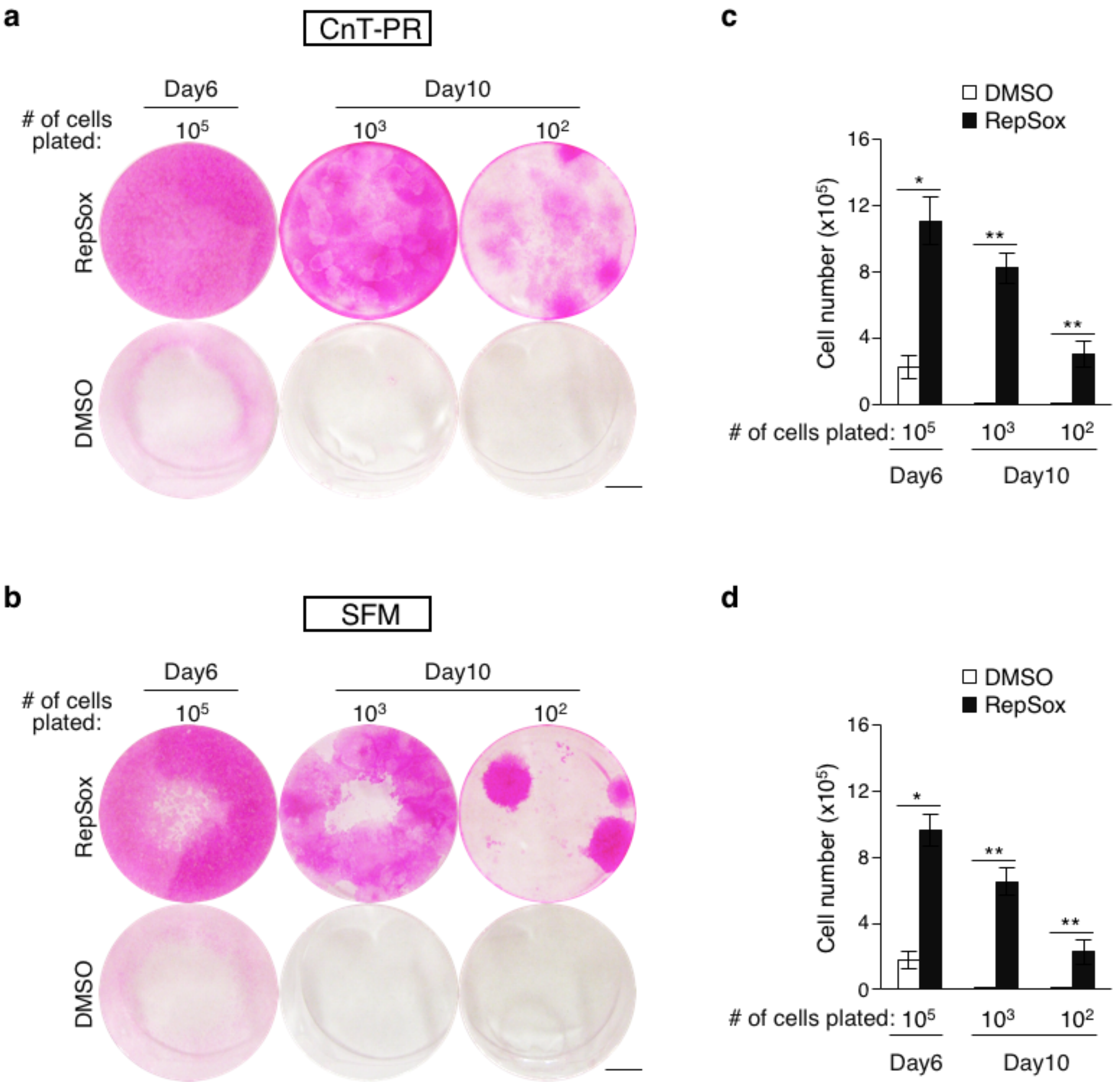

a
CnT-PR
Day6
Day10
# of cells plated:
10^5
10^3
10^2
RepSox
DMSO
b
SFM
Day6
Day10
# of cells plated:
10^5
10^3
10^2
RepSox
DMSO
c
DMSO
RepSox
Cell number ($x10^5$)
16
12
8
4
0
*
**
**
# of cells plated: 10^5 10^3 10^2
Day6
Day10
d
DMSO
RepSox
Cell number ($x10^5$)
16
12
8
4
0
*
**
**
# of cells plated: 10^5 10^3 10^2
Day6
Day10

Figure S3

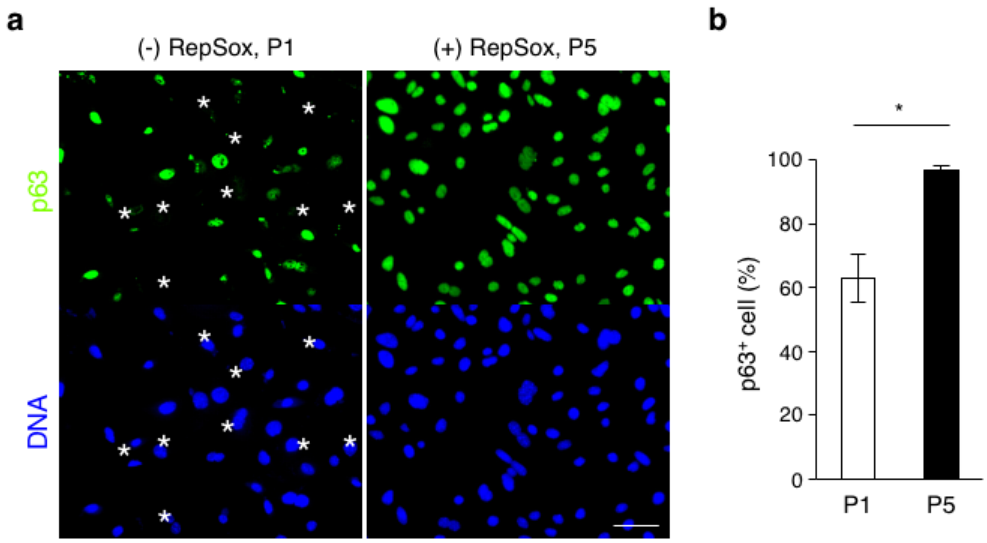

Figure S4

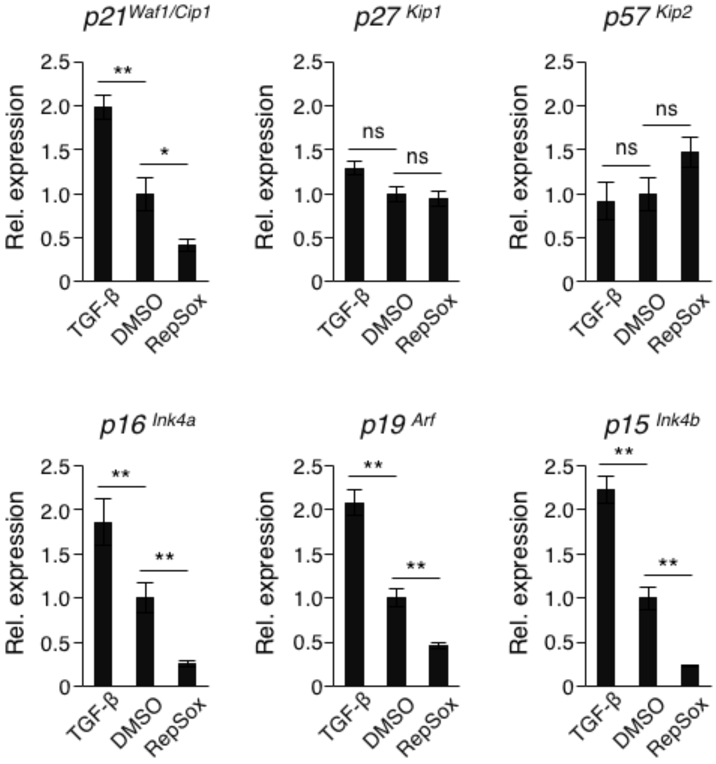

p21 Waf1/Cip1
p27 Kip1
p57 Kip2
p16 Ink4a
p19 Arf
p15 Ink4b
Rel. expression
2.5
2.0
1.5
1.0
0.5
0
TGF-β
DMSO
RepSox
**
*
ns

Figure S5

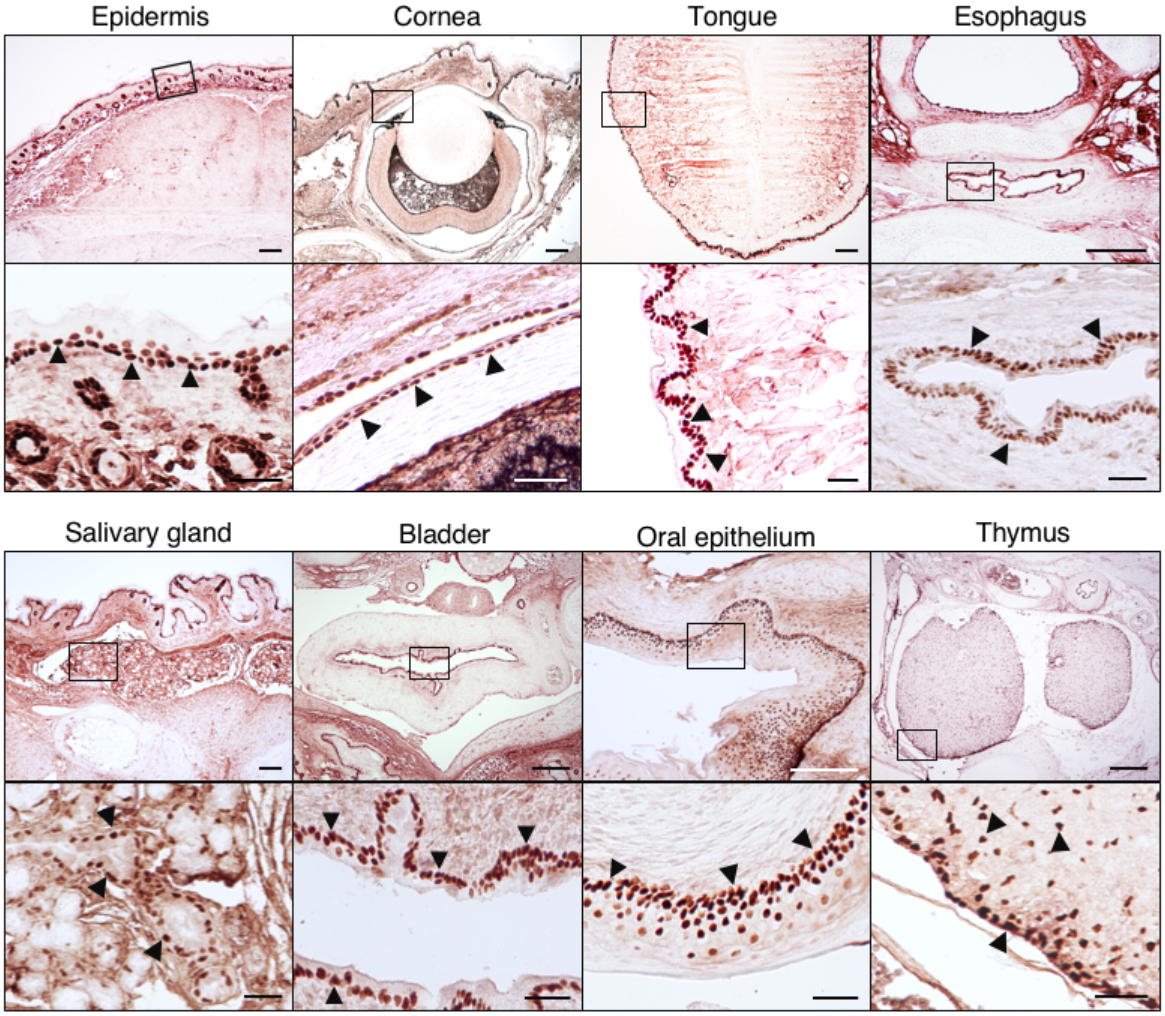

Epidermis
Cornea
Tongue
Esophagus
Salivary gland
Bladder
Oral epithelium
Thymus

Figure S6

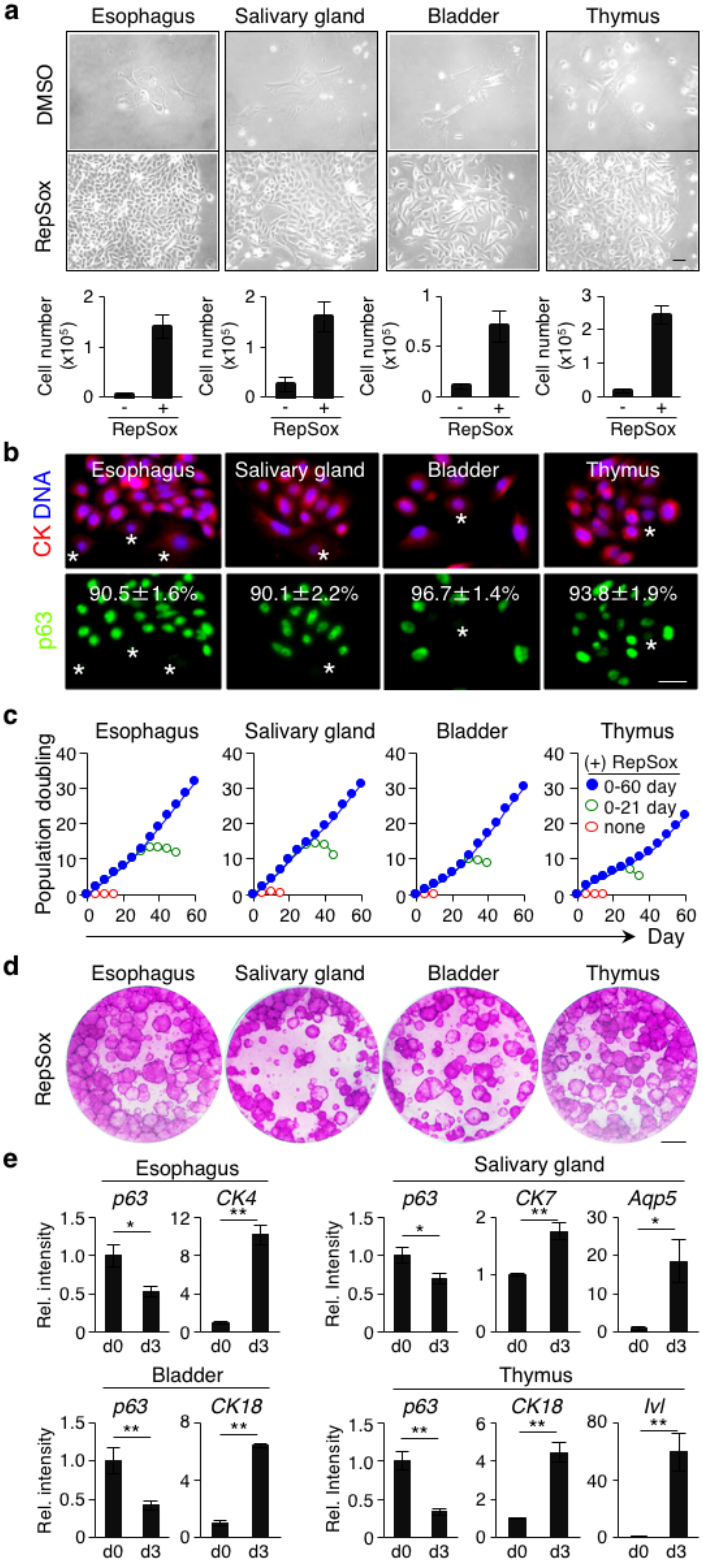

a
Esophagus
Salivary gland
Bladder
Thymus
DMSO
RepSox
Cell number (x10^5)
RepSox
b
CK DNA
p63
90.5±1.6%
90.1±2.2%
96.7±1.4%
93.8±1.9%
c
Population doubling
(+) RepSox
0-60 day
0-21 day
none
Day
d
e
Rel. intensity
p63
CK4
CK7
Aqp5
CK18
Ivl
d0
d3

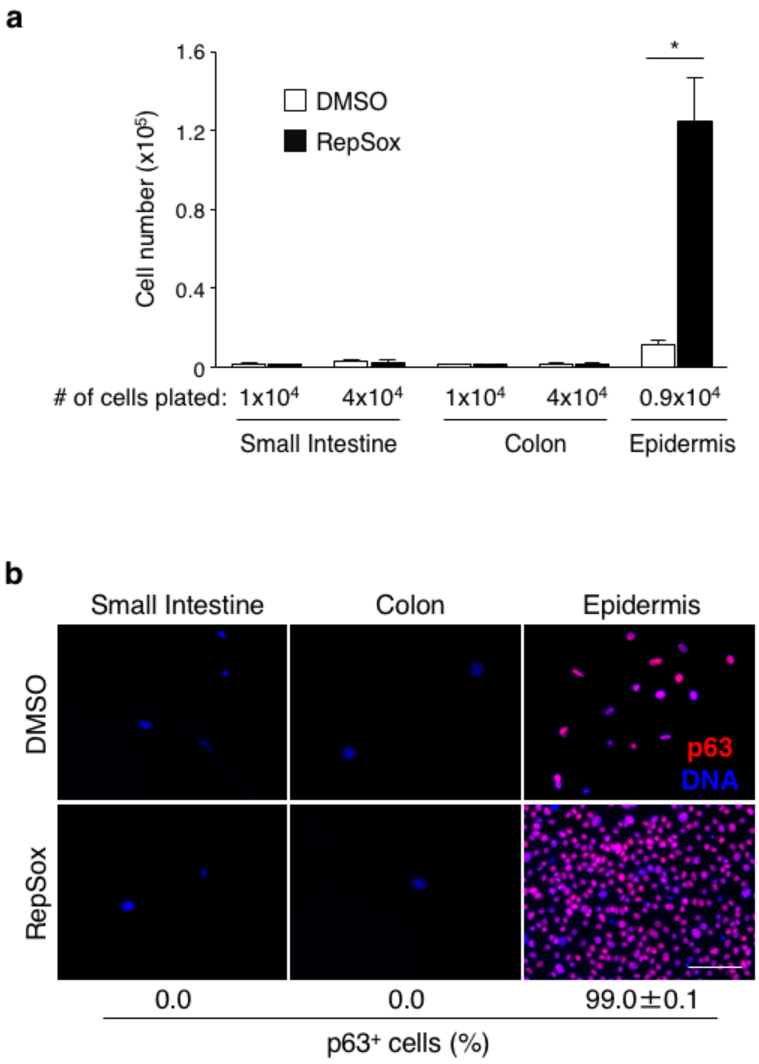
a
1.6
1.2
0.8
0.4
0
Cell number (x10^5)
DMSO
RepSox
*
# of cells plated: 1x10^4 4x10^4 1x10^4 4x10^4 0.9x10^4
Small Intestine
Colon
Epidermis
b
Small Intestine
Colon
Epidermis
DMSO
RepSox
p63
DNA
0.0
0.0
99.0±0.1
p63+ cells (%)

Figure S8

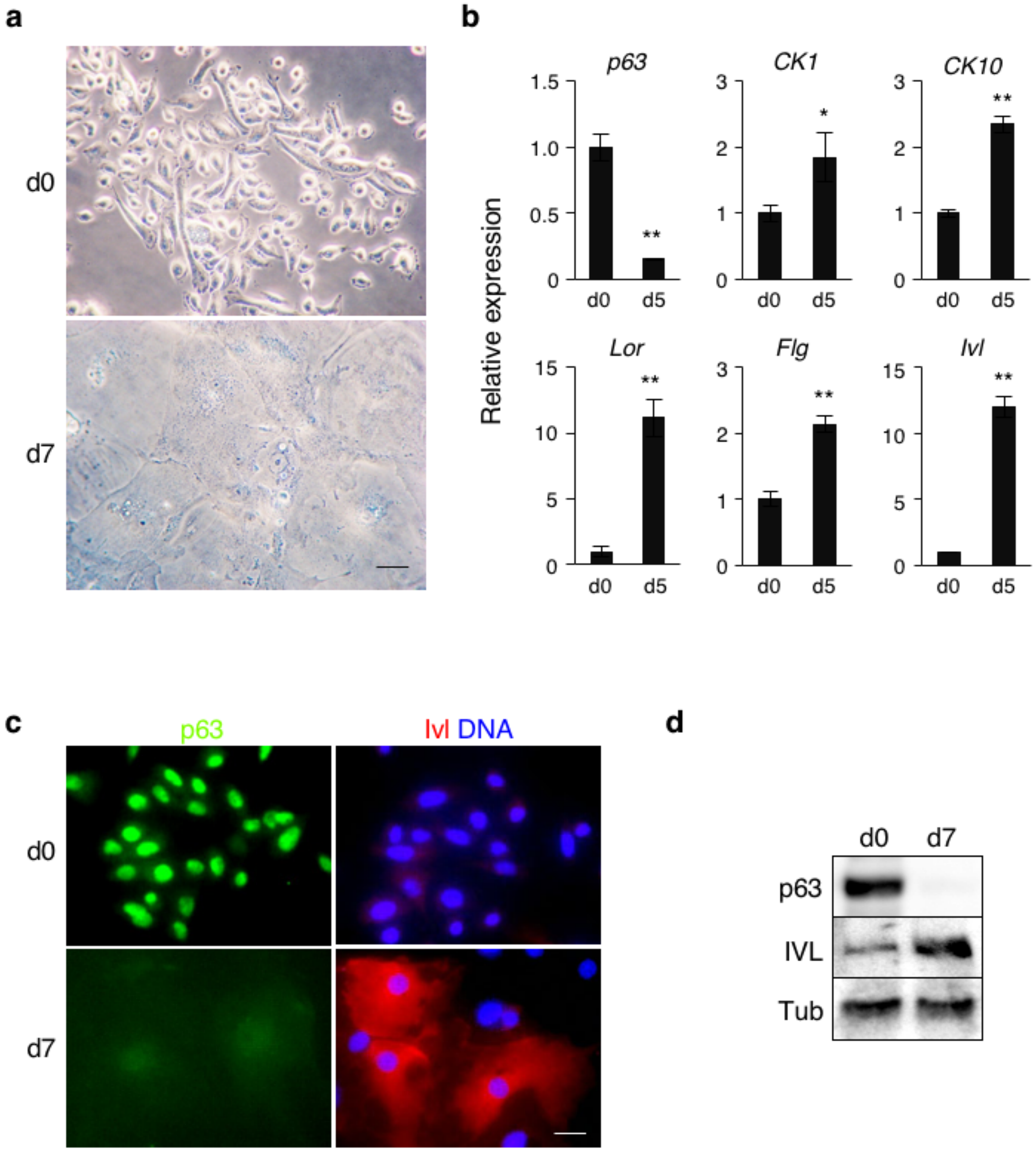

a
d0
d7
b
Relative expression
p63
CK1
CK10
Lor
Flg
Ivl
1.5
1.0
0.5
0
3
2
1
15
10
5
d0
d5
*
**
c
p63
Ivl DNA
d
p63
IVL
Tub

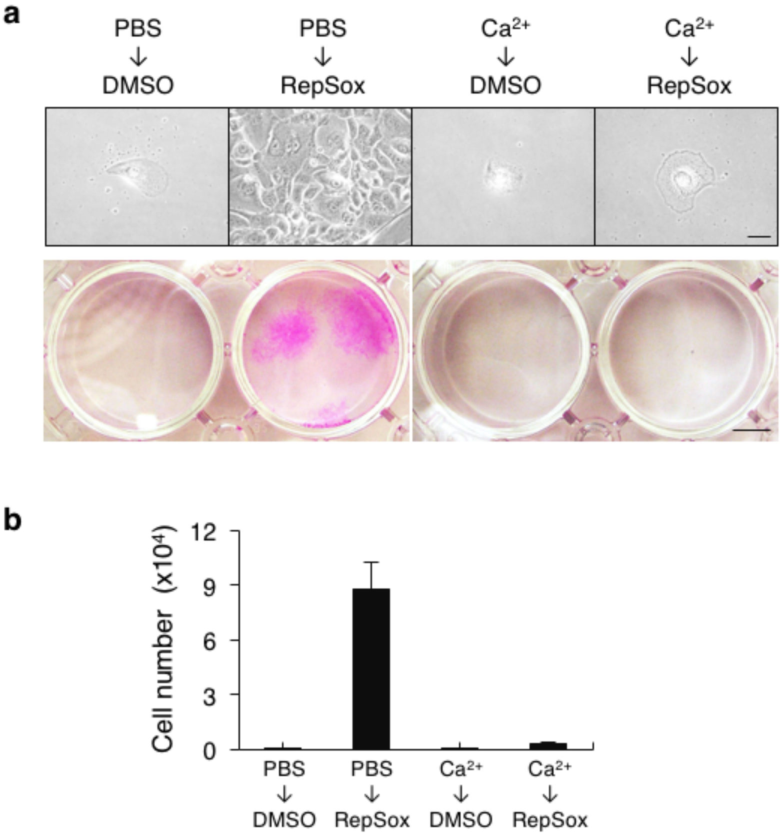
a
PBS
↓
DMSO
PBS
↓
RepSox
Ca2+
↓
DMSO
Ca2+
↓
RepSox
b
Cell number (x10⁴)
12
9
6
3
0
PBS
↓
DMSO
PBS
↓
RepSox
Ca2+
↓
DMSO
Ca2+
↓
RepSox

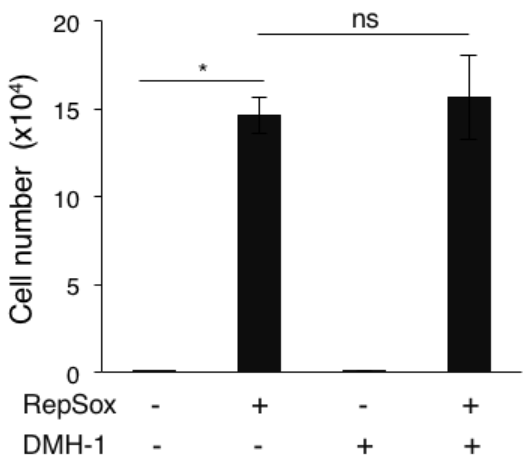
Cell number (x10^4)
20
15
10
5
0
ns
*
RepSox - + - +
DMH-1 - - + +

Figure S11

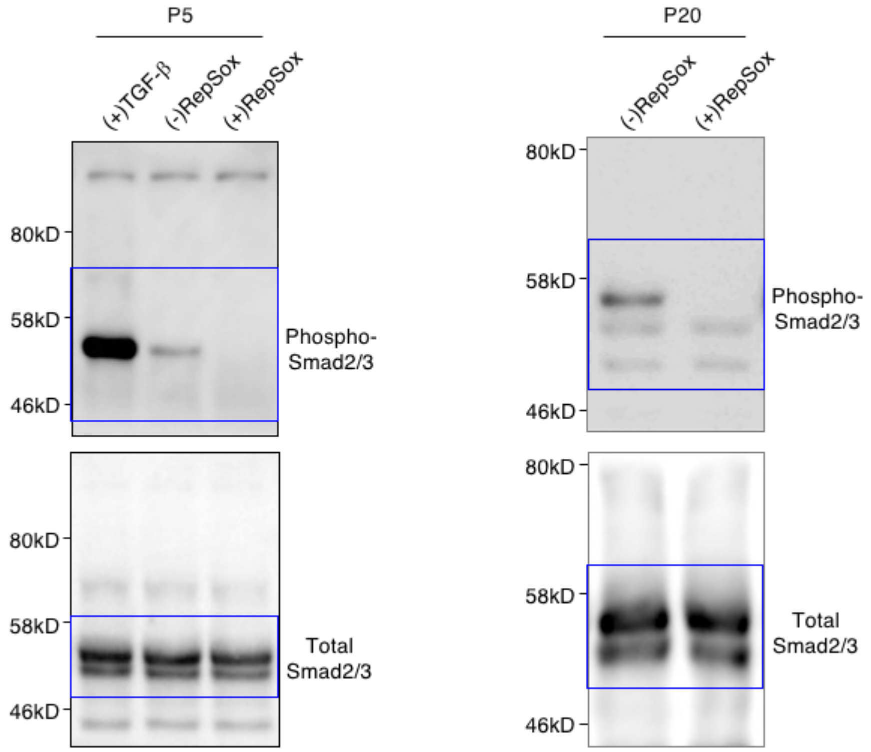

P5
(+)TGF-β
(-)RepSox
(+)RepSox
80kD
58kD
46kD
Phospho-Smad2/3
Total Smad2/3
P20

Figure S12

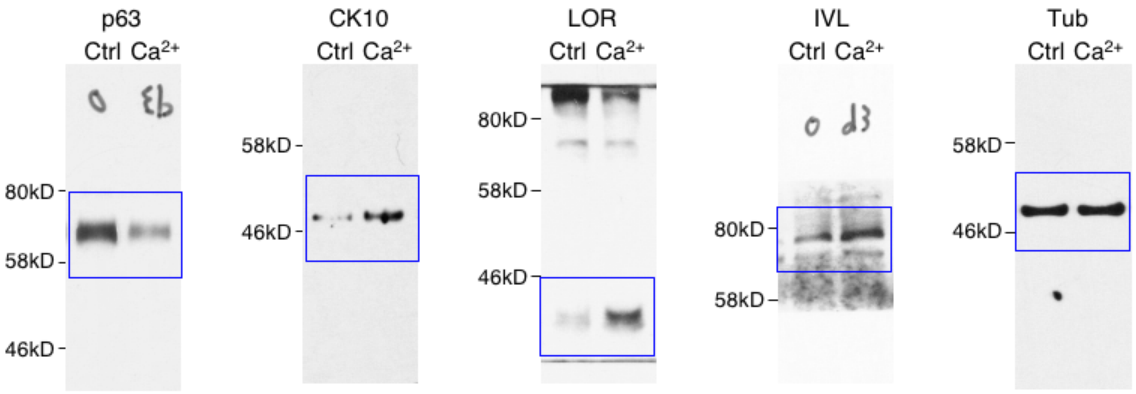

p63
Ctrl Ca2+
80kD
58kD
46kD
CK10
Ctrl Ca2+
58kD
46kD
LOR
Ctrl Ca2+
80kD
58kD
46kD
IVL
Ctrl Ca2+
80kD
58kD
Tub
Ctrl Ca2+
58kD
46kD

Figure S13

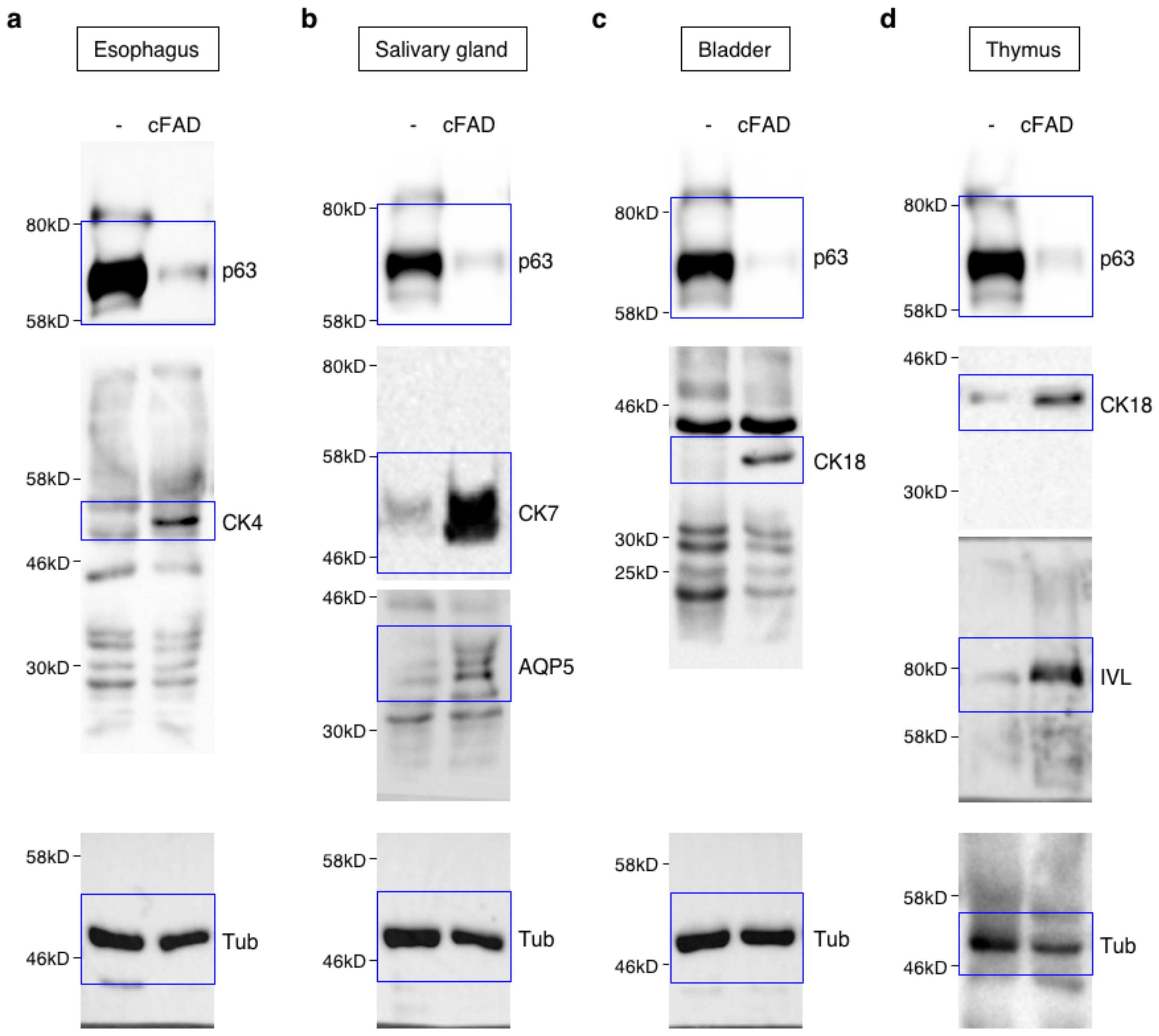

a
Esophagus
- cFAD
80kD
58kD
p63
58kD
46kD
CK4
30kD
58kD
46kD
Tub
b
Salivary gland
- cFAD
80kD
58kD
p63
80kD
58kD
46kD
CK7
46kD
AQP5
30kD
58kD
46kD
Tub
c
Bladder
- cFAD
80kD
58kD
p63
46kD
CK18
30kD
25kD
58kD
46kD
Tub
d
Thymus
- cFAD
80kD
58kD
p63
46kD
CK18
30kD
80kD
IVL
58kD
58kD
46kD
Tub

Figure S1. Growth response of mouse epidermal keratinocytes to treatment with TGF-β and BMP signaling inhibitors.

Newborn mouse-derived, CnT-PR-expanded CK^+ epidermal cells ($2x10^4$) were cultivated in CnT-PR media for 10 days in the presence of increasing concentration of TGF-β signaling inhibitors (RepSox, SB525334, LY364947, SB431542, and A83-01) or a BMP signaling inhibitor (DMH-1) as indicated. Data shown are mean±s.e.m. (n=3).

Figure S2. TGF-β signaling inhibition stimulates the growth of mouse epidermal keratinocytes in chemically-defined alternative SFM basal media.

Newborn mouse-derived, CnT-PR-expanded CK^+ epidermal cells were seeded at 10^2, 10^3 and 10^5 cells per well in 12-well plates and grown in CnT-PR (**a** and **c**) or SFM (**b** and **d**) media in the presence or absence of 1 μM RepSox, followed by Rhodamine B staining (**a** and **b**) and cell counting (**c** and **d**) at day 6 (10^5 cells) and day 10 (10^2 and 10^3 cells). Bars=5 mm. Data shown in (**c**) and (**d**) are mean±s.e.m. (n=3). *P<0.01; **P<0.005.

Figure S3. Enrichment of $p63^+$ mouse primary epidermal keratinocytes by TGF-β signaling inhibition in culture.

(**a**) Representative immunofluorescence images of mouse epidermal cells in culture, stained with anti-p63 antibodies (upper panels) and counterstained with Hoechst 33342 (lower panels). Data shown are newborn mouse-derived primary epidermal cells, grown in the absence of RepSox at P1 (left panels) or presence of 1 μM RepSox at P5 (right panels). Asterisks indicate representative $p63^-$ cells at P1. Bar=50 μm. (**b**) Quantification of (**a**). Data shown are percentage of $p63^+$ epidermal cells per field, expressed as mean±s.e.m. (n=4 for P1 and n=5 for P5). *P<0.001

Figure S4. TGF-β signaling regulates expression of the CDK inhibitor genes in mouse epidermal progenitor cells.

Quantitative RT-PCR analysis of the CDK inhibitor genes in mouse epidermal progenitor cells. Newborn mouse-derived, RepSox-expanded P5 epidermal cells were left untreated for 48 hrs in CnT-PR media, followed by cultivation in the presence of 1 ng/ml TGF-β2, 0.1% DMSO or 1 μM RepSox for 24 hrs. Data shown are normalized to the housekeeping gene *Rps18* and expressed as mean±s.e.m. (n=3). Expression in control cells (DMSO) was set to 1.0. *P<0.01; **P<0.005; *ns*, not significant.

Figure S5. Expression of p63 in various mouse epithelia.

Immunohistochemistry of newborn mouse epithelia stained with anti-p63 antibodies. Lower panels in each tissue show higher magnification of the boxed area in the upper panels. Arrowheads indicate representative p63^{+} cells in each epithelium. Bars, 250 μm (upper panels) and 50 μm (lower panels).

Figure S6. Inhibition of TGF-β signaling enables long-term expansion of p63^{+} epithelial progenitor cells of postnatal mice.

(**a**) Representative images of primary cells harvested from 4-week-old mouse esophagus, salivary gland, bladder, and thymus, grown in the presence (lower) or absence (upper) of 1 μM RepSox for 6 days. Bar=25 μm. Bar graphs below indicate the numbers of cells at day 7 in the presence or absence of 1 μM RepSox. CnT-PR-expanded P1 cells ($2x10^{4}$) were seeded. Data shown are mean±s.e.m. (n=3). (**b**) Representative immunofluorescence images of 4-week-old mouse-derived, RepSox-expanded P1 epithelial cells stained with anti-p63 and anti-pan-CK antibodies and counterstained with Hoechst 33342 (DNA). Numbers shown in lower panels represent percentages of p63^{+} cells per field, expressed as mean±s.e.m. (n=3). Bar=25 μm. (**c**) Population doubling of 4-week-old mouse-derived, RepSox-expanded P2 epithelial cells grown in CnT-PR media in the presence of 1 μM RepSox for 0, 21, and 60 days. (**d**) Representative images of Rhodamine B staining of epithelial clones grown in 3T3-J2 co-culture for 14 days in the presence of 1 μM RepSox. Four-week-old mouse-derived, RepSox-expanded P2 epithelial cells were used (n≥4). Bar=5 mm. (**e**) Quantitative RT-PCR analysis of epithelial cell differentiation marker genes, *p63, CK4, CK7, CK18*, *Ivl*, and *Aqp5* in 4-week-old mouse-derived, RepSox-expanded P2 (esophagus, salivary gland, and thymus) and P3 (bladder) epithelial cells. Data shown are normalized to the housekeeping gene *Gapdh* and expressed as mean±s.e.m. (n=3). *P<0.05; **P<0.01.

Figure S7. TGF-β signaling inhibition does not stimulate the growth of primary cells of p63-independent mouse epithelia in CnT-PR media.

(**a**) Primary cell numbers. Primary cells harvested from E19.0 mouse small intestine, colon, and epidermis were seeded as indicated and grown in the presence or absence of 1 μM RepSox for 10 days. Data shown are mean±s.e.m. (n=3). *P<0.001. (**b**) Representative immunofluorescence images of primary cells grown for 15 days in the presence or absence of 1 μM RepSox, followed by immunofluorescence staining

with anti-p63 antibodies and counterstaining with Hoechst 33342 (DNA). Numbers shown below represent percentages of $p63^+$ cells per field in culture with 1 μM RepSox, expressed as mean±s.e.m. (n=3). Bar=100 μm.

Figure S8. Differentiation of RepSox-expanded epidermal progenitor cells in monolayer culture with cFAD media.

Newborn mouse-derived, RepSox-expanded P8 epidermal cells were transferred from CnT-PR media to cFAD and grown for 5-7 days. (**a**) Representative images of epidermal cells at day 0 (upper) and day 7 (lower) of cFAD culture. Bar=40 μm. (**b**) Quantitative RT-PCR analysis of epidermal cell differentiation marker genes. Epidermal cells were induced to differentiate by treatment with cFAD for 5 days. Data shown are normalized to the housekeeping gene *Gapdh* and expressed as mean±s.e.m. (n=3). *P<0.05; **P<0.01. (**c**) Representative immunofluorescence images of epidermal cells at day 0 (upper) and day 7 (lower) of cFAD culture, stained with anti-p63 and anti-involucrin (Ivl) antibodies and counterstained with Hoechst 33342 (DNA). Bar=10 μm. (**d**) Western blot analysis using antibodies against p63 and involucrin (IVL). Epidermal cells were induced to differentiate by treatment with cFAD for 7 days. Tubulin-α (Tub) was used as a loading control.

Figure S9. Mouse epidermal cells differentiated by Ca^{2+} treatment no longer proliferate in response to TGF-β signaling inhibition.

Newborn mouse-derived, CnT-PR-expanded CK^+ primary epidermal cells ($2x10^4$) were induced to differentiate by treatment with 0.3 mM Ca^{2+} for 5 days, followed by the removal of Ca^{2+} and cultivation for additional 14 days in the presence or absence of 1 μM RepSox. (**a**) Representative images of mouse epidermal cells at a higher magnification (upper panels) and whole culture wells stained with Rhodamine B (lower panels) at the end of 14-day secondary culture. Bars, 25 μm (upper panels) and 5 mm (lower panels). (**b**) Epidermal cell numbers at the end of 14-day secondary culture, expressed as mean±s.e.m. (n=3).

Figure S10. Inhibition of BMP signaling by DMH-1 does not enhance the TGF-β inhibition-mediated increase of mouse epidermal progenitor cell growth.

Newborn mouse-derived, CnT-PR-expanded CK^+ primary epidermal cells ($2x10^4$) were cultivated in CnT-PR media in the presence (+) or absence (-) of a TGF-β signaling inhibitor (RepSox, 1 μM), a BMP signaling inhibitor (DMH-1, 1 μM), or a

combination of both (1 μM each) for 14 days. Data shown are mean±s.e.m. (n=3). *P<0.005; *ns*, not significant.

Figure S11. Full-length Western blots used in Figure 1h.
Newborn mouse-derived, RepSox-expanded P5 and P20 epidermal keratinocytes were further grown in continuous presence (+RepSox) or absence (-RepSox) of 1 μM RepSox for 24 hrs. Culture of P5 cells stimulated with 1 ng/ml TGF-β for 1 hr prior to lysis was used as a positive control. Data shown are expression of total (lower panels) and phosphorylated (upper panels) Smad2/3 as determined by Western blot. The boxed areas are shown in Figure 1h.

Figure S12. Full-length Western blots used in Figure 3c.
Newborn mouse-derived, RepSox-expanded P17 epidermal cells were induced to differentiate by the treatment with 0.3 mM Ca^{2+} for 0 (Ctrl) and 3 (Ca^{2+}) days. Western blot analysis was performed using antibodies against p63, CK10, loricrin (LOR), and involucrin (IVL). Tubulin-α (Tub) was used as a loading control. The boxed areas are shown in Figure 3c.

Figure S13. Full-length Western blots used in Figure 6.
Newborn mouse-derived epithelial progenitor cells expanded by RepSox treatment were grown in CnT-PR media in the presence of 1 μM RepSox to sub-confluence, followed by cultivation in cFAD for 7 days in the absence of RepSox. Epithelial progenitor cells used were derived from the (**a**) esophagus, (**b**) salivary gland, (**c**) bladder, and (**d**) thymus. Data shown are Western blot analysis with antibodies against p63, CK4, CK7, CK18, aquaporin 5 (AQP5), and involucrin (IVL). Tubulin-α (Tub) was used as a loading control. The boxed areas are shown in Figure 6.

Supplementary Table S1. Oligonucleotide sequence of PCR primers used in this study

Gene	Forward primer (5'-3')	Reverse primer (5'-3')
p63	TGCCCAGACTCAATTTAGTG	TGGAGCTGGGCTGTGCATAG
$p21^{Waf1/Cip1}$	GAACATCTCAGGGCCGAAAA	CAATCTGCGCTTGGAGTGAT
$p27^{Kip1}$	TCAAACGTGAGAGTGTCTAACG	CCGGGCCGAAGAGATTTCTG
$p57^{Kip2}$	GCAGGACGAGAATCAAGAGCA	GCTTGGCGAAGAAGTCGTT
$p16^{Ink4a}$	CGTACCCCGATTCAGGTGAT	TTGAGCAGAAGAGCTGCTACGT
$p19^{Arf}$	GCCGCACCGGAATCCT	TTGAGCAGAAGAGCTGCTACGT
$p15^{Ink4b}$	AGATCCCAACGCCCTGAAC	CCCATCATCATGACCTGGATT
CK1	TGGGAGATTTTCAGGAGGAGG	GCCACACTCTTGGAGATGCTC
CK4	TCGGCAGCAGAAGTCTTTACA	CAGCACCGTATCCTCCAACG
CK7	AGGAGATCAACCGACGCAC	GTCTCGTGAAGGGTCTTGAGG
CK8	TCCATCAGGGTGACTCAGAAA	CCAGCTTCAAGGGGCTCAA
CK10	GCCTCCTACATGGACAAAGTC	GCTTCTCGTACCACTCCTTGA
CK14	GAGGAGACCAAAGGCCGTTAC	GAGGAGAATTGAGAGGATGAGGA
CK18	ACTCCGCAAGGTGGTAGATGA	TCCACTTCCACAGTCAATCCA
CK19	GGGGGTTCAGTACGCATTGG	GAGGACGAGGTCACGAAGC
Loricrin	GCGGATCGTCCCAACAGTATC	TGAGAGGAGTAATAGCCCCCT
Filaggrin	ATGTCCGCTCTCCTGGAAAG	TGGATTCTTCAAGACTGCCTGTA
Involucrin	ATGTCCCATCAACACACACTG	TGGAGTTGGTTGCTTTGCTTG
Aquaporin 5	AGAAGGAGGTGTGTTCAGTTGC	GCCAGAGTAATGGCCGGAT
Foxa1	ATGAGAGCAACGACTGGAACA	TCATGGAGTTCATAGAGCCCA
Sox2	GCGGAGTGGAAACTTTTGTCC	CGGGAAGCGTGTACTTATCCTT
Rps18	CATGCAGAACCCACGACAGTA	CCTCACGCAGCTTGTTGTCTA
Gapdh	AACTTTGGCATTGTGGAAGG	CACATTGGGGGTAGGAACAC
